# Multi-integrated genomic data for *Passiflora foetida* provides insights into genome size evolution and floral development in *Passiflora*

**DOI:** 10.1186/s43897-023-00076-x

**Published:** 2023-12-18

**Authors:** Yi Zou, Jie Wang, Dan Peng, Xiaoni Zhang, Luke R. Tembrock, Jinliang Yang, Jianli Zhao, Hong Liao, Zhiqiang Wu

**Affiliations:** 1 Shenzhen Branch, Guangdong Laboratory of Lingnan Modern Agriculture, Key Laboratory of Synthetic Biology, Laboratory of the Ministry of Agriculture and Rural Affairs, Agricultural Genomics Institute at Shenzhen, Chinese Academy of Agricultural Sciences, Shenzhen, China; 2 Ministry of Education Key Laboratory for Transboundary Ecosecurity of Southwest China, Yunnan Key Laboratory of Plant Reproductive Adaptation and Evolutionary Ecology, Institute of Biodiversity, School of Ecology and Environmental Science, Yunnan University, Kunming, Yunnan 650504 China; 3 College of Agriculture, Center for Genomics and Biotechnology, Fujian Agriculture and Forestry University, Fuzhou, 350002 China; 4 Department of Agricultural Biology, Colorado State University, Fort Collins, CO 80523 USA; 5 Department of Agronomy and Horticulture, University of Nebraska-Lincoln, Lincoln, NE 68583 USA; 6 Kunpeng Institute of Modern Agriculture at Foshan, Shenzhen Branch, Guangdong Laboratory of Lingnan Modern Agriculture, Agricultural Genomics Institute at Shenzhen, Chinese Academy of Agricultural Sciences, Shenzhen, 518124 China

**Keywords:** *Passiflora foetida*, Genome assembly, Transposable elements, MADS-box, Floral organ

## Abstract

*Passiflora* is a plant genus known for its extremely distinctive and colorful flowers and a wide range of genome size variation. However, how genome characteristics are related to flower traits among *Passiflora* species remains poorly understood. Here, we assembled a chromosome-scale genome of *P. foetida*, which belongs to the same subgenus as the commercial passionfruit *P. edulis*. The genome of *P. foetida* is smaller (424.16 Mb) and contains fewer copies of long terminal repeat retrotransposons (LTR-RTs). The disparity in LTR-RTs is one of the main contributors to the differences in genome sizes between these two species and possibly in floral traits. Additionally, we observed variation in insertion times and copy numbers of LTR-RTs across different transposable element (TE) lineages. Then, by integrating transcriptomic data from 33 samples (eight floral organs and flower buds at three developmental stages) with phylogenomic and metabolomic data, we conducted an in-depth analysis of the expression, phylogeny, and copy number of MIKC-type MADS-box genes and identified essential biosynthetic genes responsible for flower color and scent from glandular bracts and other floral organs. Our study pinpoints LRT-RTs as an important player in genome size variation in *Passiflora* species and provides insights into future genetic improvement.

**Graphical Abstract:**

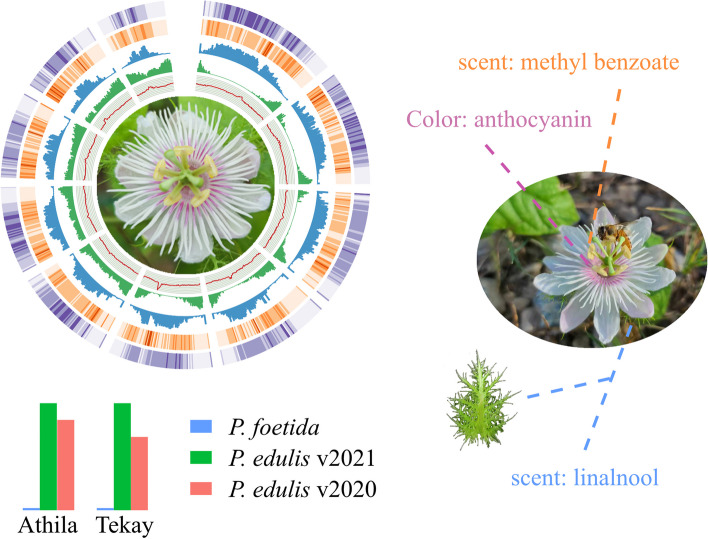

**Supplementary Information:**

The online version contains supplementary material available at 10.1186/s43897-023-00076-x.

## Core

The small genome of *Passiflora foetida* contains fewer retrotransposons than the commercial passionfruit (*Passiflora edulis*). The floral color (anthocyanins) and scent (methyl benzoate and linalool) may play important roles in its communication with pollinators and herbivores. The expression and copy number variation of MADS-box genes may underline the peculiar floral morphology of *Passiflora* species, establishing a solid foundation for future research endeavors and flower breeding programs.

## Gene & accession numbers

Sequence reads and the final genome assembly with annotation of *P. foetida* can be found in the Genome Sequence Archive in the National Genomics Data Center, China National Center for Bioinformation / Beijing Institute of Genomics, Chinese Academy of Sciences under the accession numbers PRJCA020083.

## Introduction

**Fig. 1:**
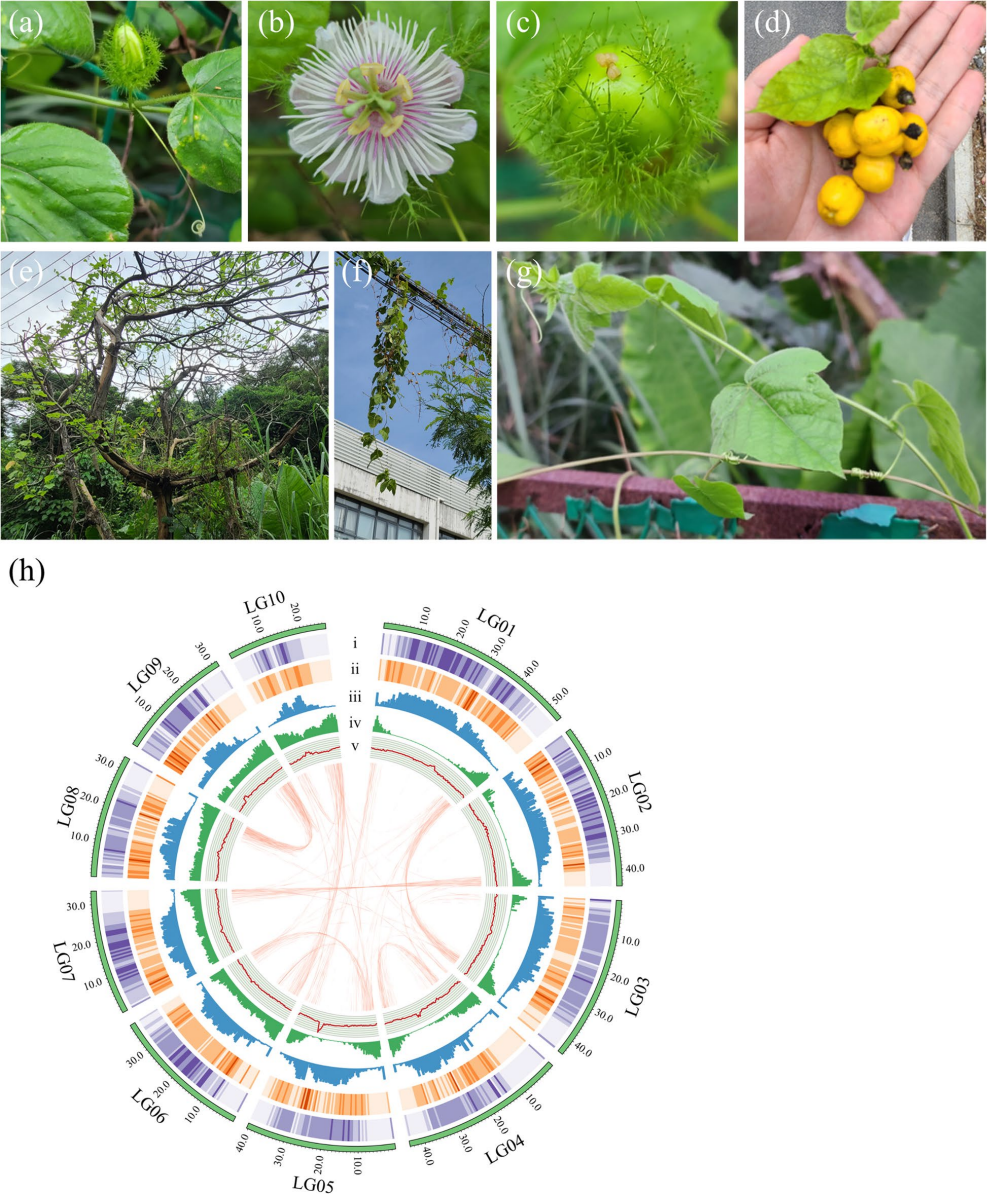
Habit and morphology of *Passiflora foetida* and its genome assembly. **a**-**g** Photos show the (**a**) flower bud surrounded by glandular bracts, (**b**) fully opened flower, (**c**) young fruit with persistent glandular bracts, (**d**) mature fruit and (**e**-**g**) examples of the climbing habit. **h** Circos plot represents the ten pseudochromosomes of *P. foetida*. Tracks represent (i) the density of Copia-LTR-RTs, (ii) the density of Gypsy-LTR-RTs, (iii) the distribution of other repeat elements, (iv) gene density, and (v) GC content. These metrics were calculated in 500 Kb windows. The arcs at the center represent collinear blocks between chromosomes containing 15 or more genes

The genus *Passiflora* contains more than five hundred species worldwide, with most of the species distributed in the Americas (Cerqueira-Silva et al. 2014). They are generally classified into five main subgenera: *Astrophaea*, *Decaloba*, *Tetrapathea*, *Deidamiodes*, and *Passiflora* (Muschner et al. 2003; Sader et al. 2019). Many species in *Passiflora* are highly valued for their edible fruits and medicinal compounds. *Passiflora edulis* is the most economically important species of the *Passiflora* genus, and it consists of two main varieties: the purple passion fruit (*P. edulis* Sims) and the yellow passion fruit (*P. edulis* f. *flavicarpa* O. Deg). The fruits of *P. edulis* possess various nutritional and medicinal compounds including amino acids, oils, flavonoids, triterpenoids, and carotenoids (He et al. 2020; Fonseca et al. 2022). *Passiflora foetida* (“Long Zhu Guo” in Chinese) is a scrambling vine native to warmer regions of the Americas and the Caribbean. However, it has also become naturalized (and in some cases invasive) in various parts of the world, including Africa, South Asia, and Hawaii. Unlike other *Passiflora* species, *P. foetida* possesses persistent bracts that surround the flowers and remain through fruiting (Fig. 1a). What is even more peculiar is that these bracts produce sticky hairs which produce digestive enzymes and thus this species is considered a protocarnivorous plant (Radhamani et al. 1995). As the epithet suggests, *P. foetida* emits a strong odor when the leaves are handled. Despite this, the fruits are edible, and the plant has been used in traditional medicine (Patil and Paikrao 2012). Chemical compositions among different *Passiflora* species vary due to differential gene expression and metabolite accumulation. For instance, sucrose is the predominant soluble sugar in *P. edulis* (de Oliveira et al. 2014), whereas it only presents in low concentrations in *P. foetida* fruits (Song et al. 2018). Thus, understanding the genomic basis behind those metabolites is urgent and would be helpful for future improvements.

In addition to edible fruits and medicinal compounds, species in *Passiflora* are well known for their exquisitely complex flowers. It has been shown that the MADS-box genes play a crucial role in floral transition and floral organ development (Ng and Yanofsky 2001). The ABCE model suggests that different combinations of MIKC-type MADS-box genes are required to specify the identities of different floral whorls (Coen and Meyerowitz 1991; Thomson et al. 2017; Shan et al. 2019). According to the model, A- and E-class genes specify sepals; A-, B-, and E-class genes specify petals; B-, C-, and E-class genes specify stamens; C- and E-class genes specify carpels; and finally, D- and E-class genes specify ovules (Rijpkema et al. 2010). The formation and development of *Passiflora* flowers exhibit peculiarities in three aspects. First, there are extra whorls between the petals and stamens, which has challenged the classical ABCE model of flower development (Classen-Bockhoff and Meyer 2016). It has been proposed that these extra whorls initiate after the formation of other whorls and might be associated with increased copies of B- and C-class genes (Classen-Bockhoff and Meyer 2016; Scorza et al. 2017). Second, it has been suggested that the development of tendrils in *Passiflora* species are modified reproductive structures and are closely connected to floral initiation and development, which are also associated with MADS-box genes (Scorza et al. 2017; Hernandes-Lopes et al. 2019). Third, the seemingly three symmetrical bracts subtending the flowers are actually composed of one bract which is the first primordium differentiated from the bud complex, and two bracteoles that originated from the axillary meristem of the first bract (Hernandes-Lopes et al. 2019). In the case of *P. foetida*, the three bracts are enlarged and highly branched with secretory glands at the branch ends, which are important for defense from and digestion of potential pests (Radhamani et al. 1995). However, for species in the *Decaloba* subgenus, the bracts are completely absent (Soares et al. 2018). Although previous studies have explored these questions at the anatomical and developmental levels, it is also important to understand the function of MADS-box genes in these processes from the functional genomic perspective to better understand how such traits evolved.

How genome sizes change is an important question in evolutionary biology with various explanations. Importantly, TEs are repetitive sequences that undergo rapid turnover and can alter the size and architecture of plant genomes (Novak et al. 2020). The activity of TEs can lead to gene inactivation, reprograming of gene expression, and with DNA recombination, they can further lead to deletion, rearrangement, and transposition of genes (Lisch 2013). LTR-RTs, a common type of plant TEs, are typically categorized into two superfamilies, Copia and Gypsy (Wicker et al. 2007). Individual lineages of LTR-RTs can vary significantly in copy numbers, even among closely related species (Stritt et al. 2020). To better understand the biological importance of LTR-RTs, it is necessary to classify them at the lineage- or clade-level and map their locations in the genome (Neumann et al. 2019; Zhang et al. 2022a, 2022b, 2022c). The known genome sizes of *Passiflora* species range from the smallest (0.212 pg of DNA C-value) in *P. organensis* to the largest (2.68 pg) in *P. quadrangularis* (Souza et al. 2004; Yotoko et al. 2011). This ten-fold genome size variation appears to be associated with flower size in the *Passiflora* subgenus but not in the *Decaloba* subgenus (Yotoko et al. 2011). Following divergence from a common ancestor (estimated to be 1.16 pg in size), the genome sizes become progressively smaller in the lineages leading to *P. foetida* (0.56 pg), while the genome sizes become progressively larger in the lineages leading to the estimated common ancestor (1.89 pg) of *P. alata* and *P. edulis* (1.26 pg) (Yotoko et al. 2011). Using the short-read dependent RepeatExplorer pipeline and fluorescent in situ hybridization, researchers found that Angela, Tekay, and Athila LTR-RT lineages are the most abundant in some *Passiflora* species, including *P. edulis*, *P. organensis*, *P. cincinnata*, and *P. quadrangularis* (Pamponet et al. 2019; Sader et al. 2021). Currently, there are only two versions of chromosome-level genomes available for *P. edulis* (Ma et al. 2021; Xia et al. 2021) and a contig-level genome of *P. organensis* belonging to the *Decaloba* subgenus (Costa et al. 2021). However, these studies use different tools to identify LTR-RTs and lack specific details on clade-level TE classification. The incomplete availability of high-quality genomes hinders a comprehensive understanding of the factors contributing to variation in chromosome architecture and genome size.

In this study, our primary goal was to generate a high-quality genome assembly of *P. foetida* using a combination of short- and long-read sequencing, as well as Hi-C scaffolding such that more accurate inferences about genome size and structure could be made. As a result, our final genome assembly was 424.18 Mb in size and included the annotation of 30,584 protein-coding genes and 509,131 TEs. With this genome assembly and annotation, we analyzed the genome evolution and whole-genome duplication events of *P. foetida* in comparison to *P. edulis*. Our findings suggest that rapid removal of LTR-RTs in *P. foetida* and elevated insertion activity of LTR-RTs in *P. edulis* contributed to the difference in genome sizes between these species. Based on a combination of phylogenetic, transcriptomic, metabolomic and genome-wide analyses, we also studied genes related to specification of floral organ identity, pigments biosynthesis, and emission of volatile compounds in *P. foetida*. This work substantially improves our understanding of the formation of the complex floral organs in *Passiflora* and provides an important resource for further research and breeding.

## Results

### Genome assembly and annotation

*Passiflora foetida* is a perennial scrambling vine that belongs to the Passifloraceae family in Malpighiales order. The small (3 ~ 4 cm) flowers of *P. foetida* have white petals with a pink or pale purple filamentous crown (Fig. 1b). The flowers and fruits of *P. foetida* are protected by three hairy bracts that can trap small insects (Fig. 1a-c). The mature fruits are yellowish-orange and usually ~ 2 cm in diameter with hard black seeds (Fig. 1d). The tendril-aided climbing behavior of *P. foetida* allows the plant to climb trees, cables, fences, and other structures (Fig. 1e-g).

We sequenced and assembled the genome of *P. foetida* using a combination of short reads, Nanopore long reads, and Hi-C scaffolding (Additional file 2: Table S1). We first used Kmerfreq to estimate the size, repetitiveness, and heterozygosity of the *P. foetida* genome based on 25.70 Gb of Illumina paired-end reads. The result indicated an estimated genome size of 465.46 Mb with a high repeat content and low heterozygosity (Additional file 1: Fig. S1a). We then generated 62.15 Gb of Nanopore single-pass reads and employed NextDenovo and NextPolish to achieve a de novo genome assembly of 429.16 Mb with high contiguity (607 contigs, N50 = 13.06 Mb). After removing 36 mitochondrial/plastid contigs, we further used Hi-C data (87.50 Gb filtered Illumina paired-end reads) to group the remaining contigs into ten pseudochromosomes using LACHESIS. The final Hi-C assembly we used for downstream analysis was 391.01 Mb for ten pseudochromosomes (LG01 to LG10) and 33.17 Mb for unplaced contigs (Contig1 to Contig344; Fig. 1h and Additional file 1: Fig. S2; Additional file 2: Table S2). The final assembly exhibited high mapping ratios of both short (99.63%) and long reads (99.39%), high completeness (98.36%) and consensus sequence accuracy (QV = 28.72, error rate = 0.0013) using Merqury, nearly no contamination using Blobtools (Additional file 1: Fig. S1b), very few redundant sequences using purge_haplotigs (Additional file 1: Fig. S1c), as well as Benchmarking Universal Single-Copy Orthologs (BUSCO; Simao et al. 2015) with a value of 98.76%.

We used the EDTA pipeline to annotate the TEs in the genome of *P. foetida*, revealing that the genome was made up of 66.71% identifiable repeats. The LTR assembly index (LAI) indicated a high level of genome contiguity (LAI = 18.44) of our assembly. By combining transcript-based, homology-based and ab initio prediction results with EVM and the PASA pipeline, we identified a total of 30,584 protein-coding genes in the genome of *P. foetida*, with a BUSCO completeness of 94.2%. Furthermore, by searching against the SwissProt, (Gene Ontology) GO, Kyoto Encyclopedia of Genes and Genomes (KEGG), Pfam, and NR databases, we found that 97.07% of these genes were functionally annotated by at least one database (Additional file 2: Table S3). In addition, noncoding RNAs (ncRNAs) were comprehensively annotated in our study, including 1129 rRNAs, 1174 tRNAs, 1229 snRNAs, and 144 miRNAs (Additional file 2: Table S4). In summary, we successfully generated a well-annotated genome assembly of *P. foetida* with high contiguity and completeness (Additional file 1: Fig. S1h), which served as the basis for downstream analysis.

### Genome evolution and whole-genome duplication

**Fig. 2:**
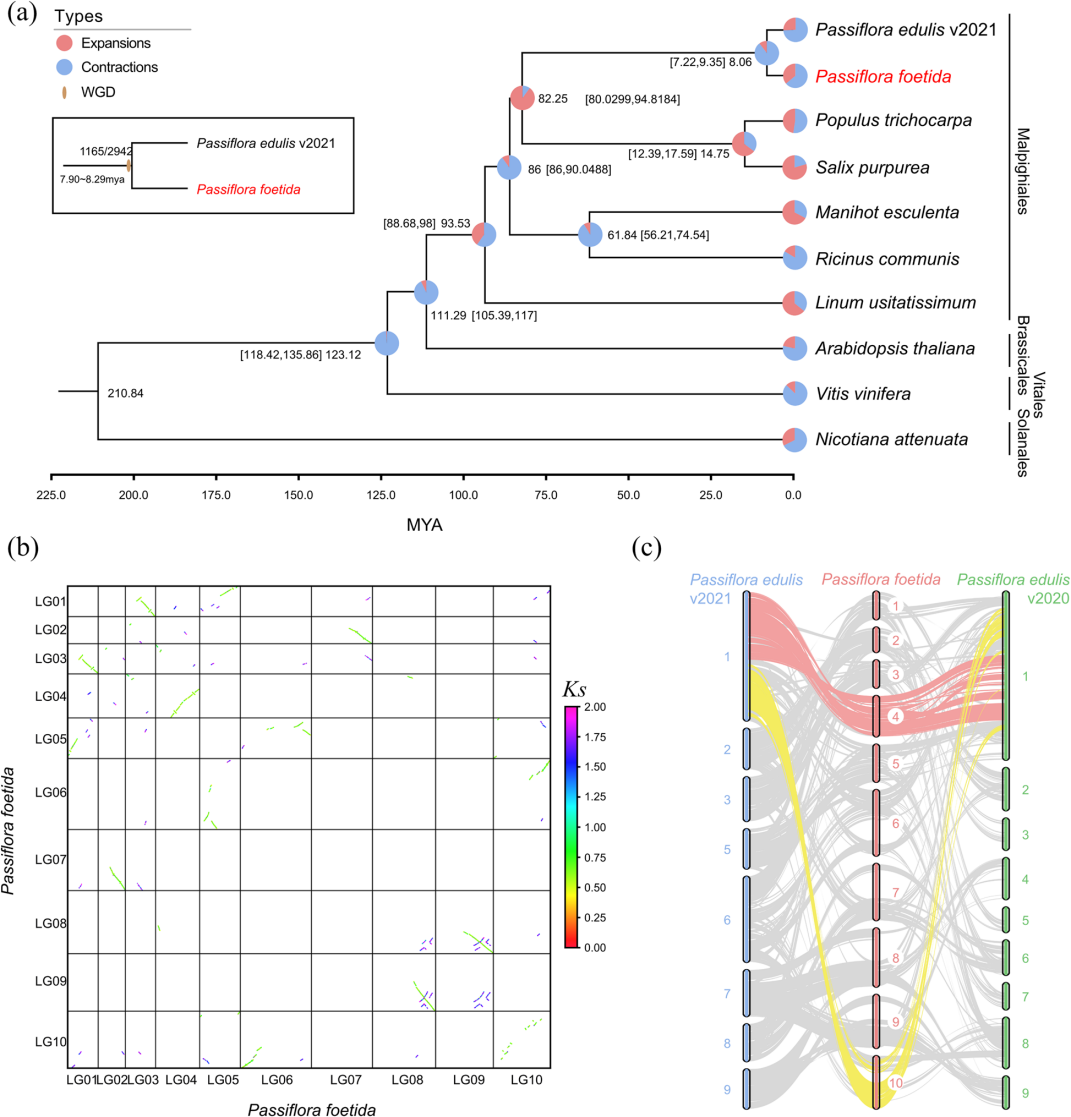
Evolutionary analysis of *P. foetida*. **a** A phylogenetic tree of ten species including *P. foetida* is shown based on 278 single-copy genes. Expanded and contracted gene families and divergence times are shown at tips and nodes. **b** A dot plot shows paralogous genes in *P. foetida*. A recent whole genome duplication event (green dots with lower *Ks* values) and an ancient whole genome triplication event (purple dots with higher *Ks* values) could be recognized. **c** Syntenic blocks of paralogous genes between *P. foetida* and two versions of *P. edulis*

To investigate the genome evolution of *P. foetida*, we conducted a comparative analysis with the genomes of nine species, including six Malpighiales species (*P. edulis*, *Salix purpurea*, *Populus trichocarpa*, *Manihot esculenta*, *Ricinus communis*, *Linum usitatissimum*, and three other angiosperm species *Arabidopsis thaliana*, *Vitis vinifera*, and *Nicotiana attenuata*). In total, we identified 17,787 gene families, among which 278 were found to be single-copy gene families. We constructed a phylogenetic tree based on these single-copy gene families and estimated the divergence time between *P. foetida* and *P. edulis* to be approximately 8.96 mya, which aligned closely with the result estimated from the TimeTree (Kumar et al. 2017) database (Fig. 2a). We next employed CAFE to analyze the expansion and contraction of gene families. The results showed that there were 117 expanded and 47 contracted gene families in *P. foetida* relative to its common ancestor with *P. edulis* (Fig. 2a). GO and KEGG enrichment analysis indicated that the expanded gene families were enriched in “ion binding” and “transcription factors”, while the contracted gene families were enriched in “immune system process”, “Ras signaling pathway”, and “terpenoid biosynthesis” in *P. foetida* (Additional file 1: Fig. S3; Additional file 3: Table S5 and S6).

To analyze potential whole-genome duplication (WGD) events, we initially identified paralogous genes within syntenic blocks identified by MCScanX. By calculating the synonymous nucleotide substitution rate (*Ks*) of these genes, we observed a single peak for both *P. foetida* and *P. edulis* (Additional file 1: Fig. S4a), suggesting one recent WGD event shared by both species. After calibrating with the estimated divergent time, we estimated that this WGD event shared by *P. foetida* and *P. edulis* likely occurred 7.90–8.29 mya (million years ago) prior to their divergence (Fig. 2a). We also generated dot-plots of these paralogous genes within *P. foetida*, which further supported one recent WGD event and one ancient whole genome triplication (WGT) event (Fig. 2b, Additional file 1: Fig. S4b,c). Moreover, synteny analysis of the *P. foetida* genome assembly (2n = 20) with two versions of previously published *P. edulis* genome assemblies (2n = 18) revealed the correspondence of *P. edulis* Chr1 to Chr4 and Chr10 of *P. foetida*, which was possibly the result of a chromosome fission event according to the chromosome number known for this subgenus (Fig. 2c; Sader et al. 2019). Together, these results provide useful insights into the genome evolution of *P. foetida* in comparison with the congener *P. edulis*.

### Comparative analysis of genome size

**Fig. 3:**
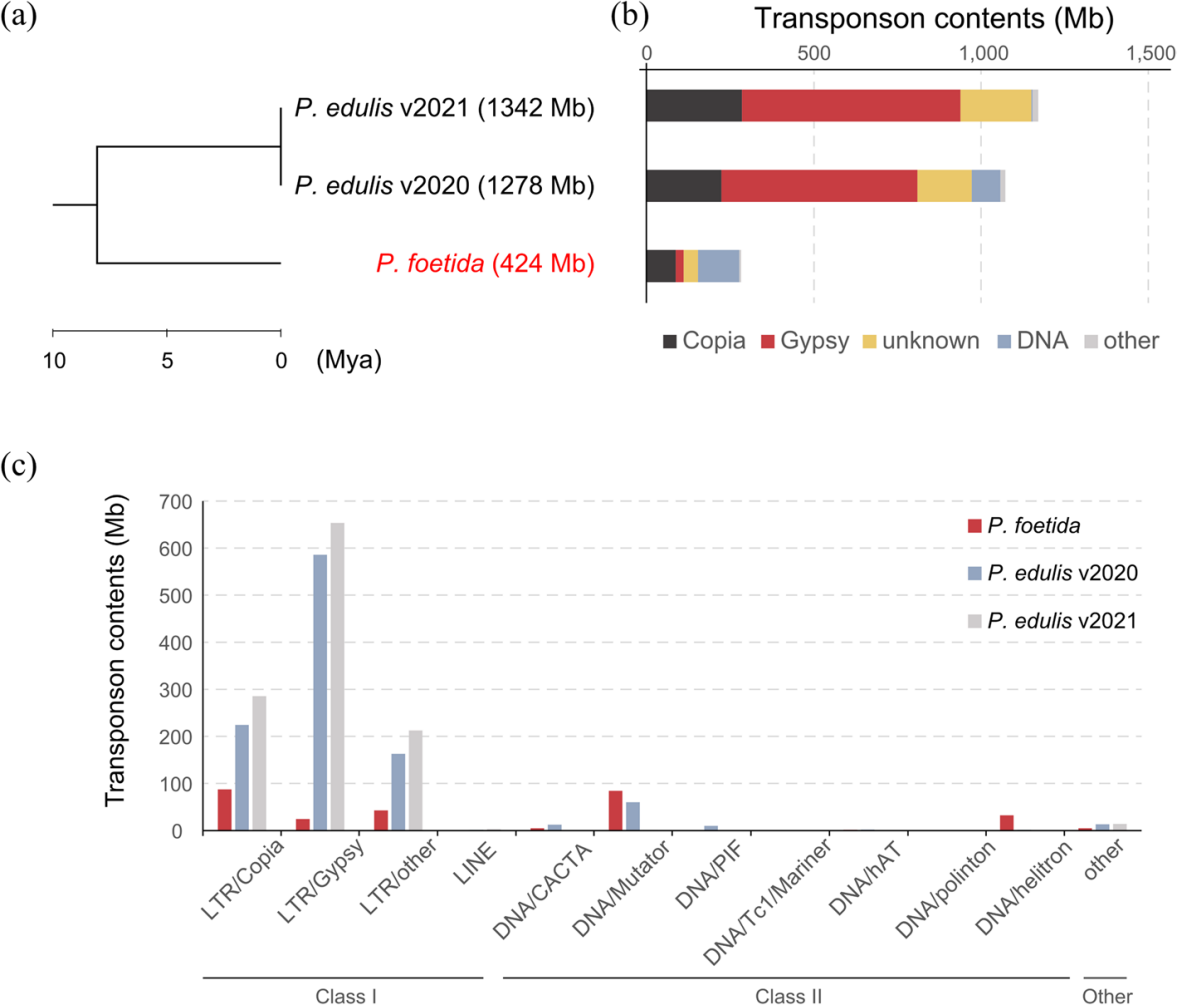
The transposable element content in *P. foetida* and *P. edulis*. **a** A tree of *P. foetida* and two *P. edulis* assemblies with genome sizes in parentheses. **b** Stacked bar plot shows the proportions of major groups of transposable elements in *P. foetida* and *P. edulis*. **c** Histogram shows the total length of different classes of transposable elements in each genome

As *P. foetida* and *P. edulis* did not undergo independent WGD events after their divergence, the large difference in genome sizes may be attributed to the differential proliferation of TEs in *P. edulis*, and/or more rapid elimination of TEs in *P. foetida*. To address these questions, we first re-annotated the TEs in the two available *P. edulis* genomes (Ma et al. 2021; Xia et al. 2021). The result indicated that the total proportion of TEs was smaller in *P. foetida* (66.71%, 282.9 Mb out of 424.2 Mb) than *P. edulis* (83.94%, 1072.9 Mb out of 1278.1 Mb and 87.29% 1171.2 Mb out of 1341.7 Mb for the two published versions; Additional file 4: Table S7). Furthermore, our analysis revealed the number of LTR-RTs was considerably larger in *P. edulis* compared to *P. foetida*, with Gypsy-type LTR-RTs showing the most significant difference (Fig. 3). For instance, we extracted a syntenic block (Additional file 1: Fig. S5b) containing 10 genes both upstream and downstream of LG10.207 in *P. foetida* and observed a much smaller number of LTR-RTs in *P. foetida* (14 copies, 4502 bp total length) than in *P. edulis* (176 copies, 173,687 bp total length in BXG2020, and 465 copies, 595,948 bp total length in BXG2021). Thus, it appeared that the homologous gene of LG10.207 in BXG2020 (P_edulis060016494.g; Additional file 1: Fig. S5a) had undergone transposition to a different chromosome, and several other genes in BXG2021 within this microsyntenic region had been transposed from elsewhere (Additional file 1: Fig. S5b). This indicates that transposition of LTR-RTs could change the order and chromosomal location of genes, potentially providing opportunities for functional remodeling.

**Fig. 4:**
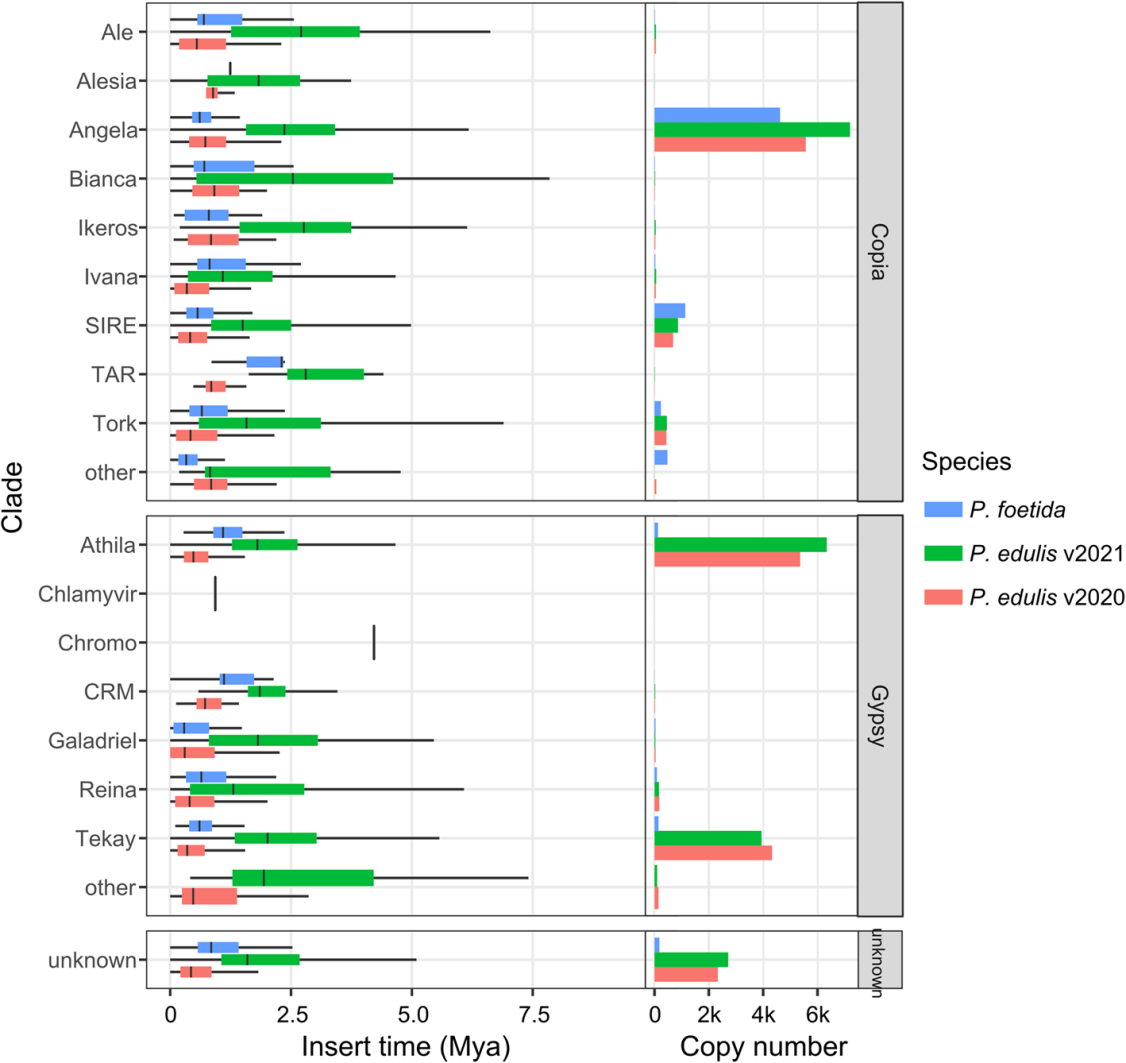
Clade-level insertion times and copy numbers of LTR-RTs in *P. foetida* and *P. edulis*. Box plots (left panel) show the distribution of inferred insertion times of all LTR-RTs for each clade. Bar plots (right panel) show clade-level copy numbers of all LTR-RTs in *P. foetida* and *P. edulis*

We then performed clade-level classification of all the EDTA-annotated LTR-RTs with TEsorter against the REXdb database of transposable element protein domains (Neumann et al. 2019; Zhang et al. 2022a), which successfully classified most LTR-RTs in *P. edulis* and *P. foetida* (Additional file 4: Table S8 and S9). We found that the two species exhibited varying copy numbers for different clades of LTR-RTs (Fig. 4). Among the high-copy LTR-RTs, two Copia clades (Angela and Tork) and three Gypsy clades (Athila, Tekay and Reina) have thousands more copies in *P. edulis* than in *P. foetida*, while only one Copia clade (SIRE) had more copies in *P. foetida* (Additional file 4: Table S9). For intermediate-copy-number LTR-RTs, three Copia clades (Ale, Ikeros and Ivana) had several hundred more copies in *P. edulis* than *P. foetida*, while two Copia clades (Bianca and TAR) and two Gypsy clades (CRM and Galadrield) had only around one hundred more copies in *P. edulis* (Additional file 4: Table S9). Interestingly, for LTR-RTs with complete and ordered domains (predicted by TEsorter), the Copia clade SIRE again had fewer copies in *P. foetida*. This indicates that SIRE copies have been removed much faster in *P. foetida*. To confirm this finding, we analyzed solo/intact LTR-RTs ratios for each clade of LTR-RTs in *P. foetida* and *P. edulis* and found that the high-copy clades Angela, SIRE, and Athila (except Tekay) had much higher ratios in *P. foetida* than *P. edulis* (Additional file 4: Table S10). The larger proportions of non-intact LTR-RTs in *P. foetida* indicates accelerated purging of LTR-RTs, which is consistent with a previous study on the changes of DNA C-values in these *Passiflora* lineages (Yotoko et al. 2011). Furthermore, we calculated the insertion time of LTR-RTs and observed that, for each clade of LTR-RTs, the average insertion time was more recent in *P. foetida* than in *P. edulis* (BXG2021; Fig. 4). The inconsistency observed in *P. edulis* (BXG2020) might be partially attributed to low assembly continuity (N50 = 70 kb), which can result in reduced assembly quality of repeat regions. Taken together, our analysis suggested that genome size difference between *P. foetida* and *P. edulis* is primarily due to the expansion of Angela, Athila, and Tekay LTR-RT lineages in *P. edulis* and more rapid elimination of the of Angela and Athila LTR-RT lineages in *P. foetida*.

### Transcriptomic analysis of different floral organs in *P. foetida*

To better understand the genetic basis underlying the unique flowers in *P. foetida*, we analyzed the transcriptomic patterns of different floral organs. We first gathered a total of 172 fully open flowers and carefully dissected each flower into eight distinct parts based on floral structures. Starting from the outer whorls to the inner ones, the flowers of *P. foetida* consist of three pinnatifid bracts (Br), five green-white petaloid sepals (Se), five white petals (Pe), two whorls of corona filaments (with white outer radii (Ri) and the pigmented inner radii), four rows of very short pali (Pa), several inner structures (the nectary, operculum, limen, annulus), the androgynophore (Ag) column, five stamens (St), and one compound pistil (Pi) composed of three to four carpels (Additional file 1: Fig. S6a-d). The pali (Pa) and the inner structures located in the receptacle cup were collected as a single sample. We performed RNA-seq analysis on each of these samples/whorls (each with three biological replicates).

From these data, we compared the differentially expressed genes (DEGs) between each pair of samples (Additional file 5: Table S11 and Additional file 6: Table S12). We defined the specifically highly expressed genes (SHEGs) for each sample group as the shared upregulated DEGs compared with the other sample groups (Additional file 1: Fig. S7; Additional file 7: Table S13). In the SHEGs of the *Passiflora*-specific radii (Ri), we found a *APETALA2* (*AP2*) transcription factor (LG05.978) with a high-expression level. This gene, namely *WRINKLED1* (*WRI1*), was originally shown to regulate oil accumulation in the seeds, and it could also regulate root auxin homeostasis and activate terpene biosynthesis through binding to AW-boxes in the promoters of target genes (Maeo et al. 2009; Kong and Ma 2018). We also found the numbers of SHEGs were among the smallest in sepals (Se) and petals (Pe), and the number of DEGs was also the lowest between sepals (Se) and petals (Pe) (Additional file 1: Fig. S7; Additional file 7: Table S13), which was consistent with the petal-like appearance of the sepals. Conversely, the numbers of SHEGs were the largest in bracts (Br), stamens (St), and pistils (Pi) (Additional file 1: Fig. S7; Additional file 7: Table S13), which may be important for the specific physiological or developmental functions of these floral organs. GO and KEGG enrichment analyses for these SHEGs showed some interesting terms in samples of stamens and pistils (Additional file 1: Fig. S8 and S9; Additional file 7: Table S14 and S15). Thus, we further performed enrichment analyses for DEGs between these two sample groups and found that the upregulated DEGs in pistils (Pi) were enriched in “cellular nitrogen compound metabolic process”, “ribosome biogenesis” and “ribonucleoprotein complex biogenesis”, while the upregulated DEGs in stamens (St) were enriched in “cellular catabolic process” and protein post-translational modifications such as “protein polyubiquitination”, “protein ubiquitination” and “protein modification by small protein conjugation” (Additional file 1: Fig. S10; Additional file 7: Table S16–19). These GO terms are similar to the study in female flowers of *Populus balsamifera*, which showed sex-biased expression of metabolic genes (Sanderson et al. 2019). Some of these enriched terms in the greener female floral organ might also suggest similar energetics. Due to the green color of the bracts (Br), most SHEGs of the Br are also related to photosynthesis. Besides, we also identified an HD-ZIP IV transcription factor *GLABRA2* (*GL2*; LG08.3456). In *Arabidopsis*, *AtGL2* was reported to play an important function in the development of leaf trichomes and root hairs (Rerie et al. 1994; Di Cristina et al. 1996). This *P. foetida GL2* homolog might play similar roles in the development of the abundant glandular hairs used to trap insects found on the bracts, but additional experiments are required to confirm this hypothesis. Carnivorous plants invest lots of genes and resources to attract, capture, digest insects and to absorb the nutrients (Fukushima et al. 2017). Interestingly, we found a homolog of *AMMONIUM TRANSPORTER 1* (*AMT1*, LG05.214) in the SHEGs of Br, which is a pitcher-predominant transporter gene and carnivory-related gene shown in two previous studies on the pitcher plant *Cephalotus* and *Dionaea muscipula*, respectively (Scherzer et al. 2013; Fukushima et al. 2017). Other carnivorous genes including genes encoding chitinases, proteinases, etc., were not identified because they were also expressed in other floral organs or because these genes might be induced after capturing the pests (Saul et al. 2023).

**Fig. 5:**
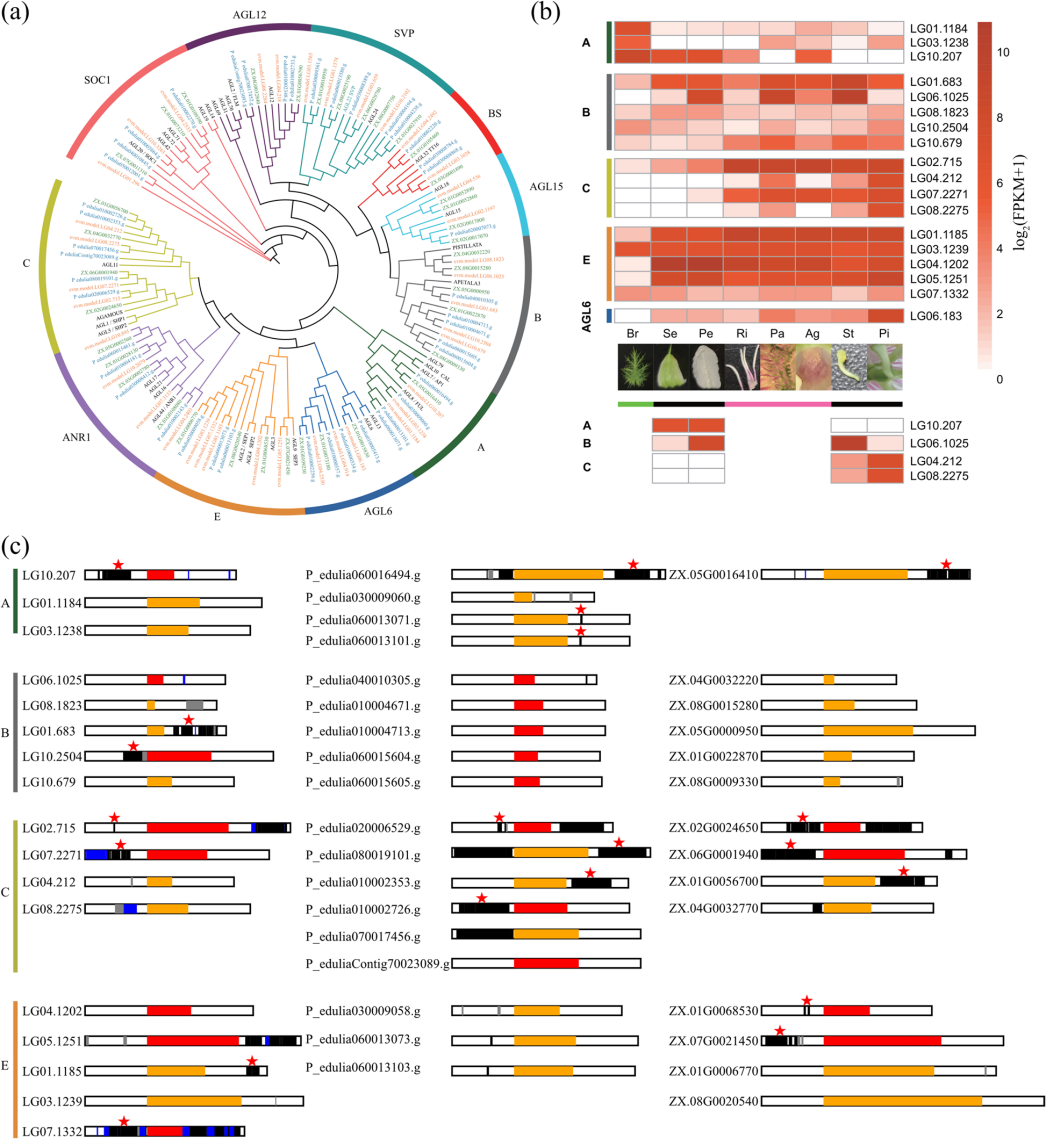
Phylogenetic and gene expression analysis of MADS-box genes in *P. foetida*. **a** A phylogenetic tree of Type-II MADS-box genes in *P. foetida* (orange), *P. edulis* (green for BXG2020, blue for BXG2021) and *Arabidopsis thaliana* (black). **b** Gene expression levels of Type-II MADS-box genes in bracts (Br), sepals (Se), petals (Pe), outer and inner radii (Ri), pali (Pa), androgynophore (Ag), stamens (St), and pistils (Pi). The bars below the photos of floral organs indicate the glandular bracts (blue), the classical four whorls (black), and the extra whorls (pink). The copies exhibiting classical expression patterns in the classical ABC model are shown below the bars. **c** Plots show transposable elements (TEs) in the 5-kb flanking regions of the ABCE MADS-box genes. Red and orange boxes indicate the genes are in forward or reverse orientation, respectively. Different types of TEs are indicated by black (Class I), blue (Class II), and gray (other) boxes. Genes with LTRs in the promoter (upstream) regions are indicated by red stars

Type-II MADS-box genes play a central role in controlling flowering time, floral meristem identity and floral organ identity (Ng and Yanofsky 2001). Copy number variation and the expression of these genes have contributed to the diversification of flower morphology in various species (Hsu et al. 2021; Hu et al. 2021; Zhang et al. 2022b). To understand the role of Type-II MADS-box genes in flower development of *P. foetida*, we conducted a combined analysis of phylogenetic, transcriptomic, and genomic data (Fig. 5 and Additional file 7: Table S20). We first constructed a phylogenetic tree of MADS-box genes with *P. foetida*, *P. edulis*, and *A. thaliana* (Fig. 5a). Among them, we identified 37 Type-II MADS-box genes in *P. foetida*. Notably, there are three copies of A-class genes (LG01.1184, LG03.1238, LG10.207) in *P. foetida*. The expression of all three A-class genes was found to be high in the bracts (Br), with one homolog (LG10.207, *AP1*) also expressed in sepals (Se) and petals (Pe) (Fig. 5b). We found five copies of B-class and four copies of C-class genes in *P. foetida*. Interestingly, an earlier study suggested that the expansion of B-class and C-class genes in *P. caerulea* was important for the formation of additional whorls between petals and stamens, with B-class genes being expressed at relatively lower levels than C-class genes in the radii (Ri) (Hemingway et al., 2011). In this study, we found that the expression of B-class genes was high in petals (Pe), pali (Pa), and stamens (St) but was low in radii (Ri), with one homolog (LG01.683) also expressed in sepals (Se) (Fig. 5b), which could be associated with its petal-like appearance (Baum and Whitlock 1999). This expression pattern was consistent with an earlier report where the expression of *PISTILLATA* (*PI*) was experimentally verified to be the highest in petals and stamens (Scorza et al. 2017). The expression of C-class genes was found to be high from radii (Ri) to the inner whorls (Fig. 5b). Therefore, the expression pattern of B-class and C-class genes in radii (Ri) of *P. foetida* is in line with that of *P. caerulea*, which implies a conserved mechanism underlying radii (Ri) development in different species of *Passiflora*. As expected, E-class genes exhibited widespread expression in all floral organs (Fig. 5b). Considering the differential expression patterns of these Type-II MADS-box genes, we further analyzed the promoter sequence of these genes, and we found that the patterns of TE distribution in the promoter regions of these genes varied greatly between different copies within each genome and among different genomes (Fig. 5c), which could potentially contribute to the modification and coordination of their expression levels. Together, our results support the importance of Type-II MADS-box genes in specifying the floral organ identity of *P. foetida* flowers, including the extra whorls.

### The expression of color- and scent-related genes in flowers

**Fig. 6:**
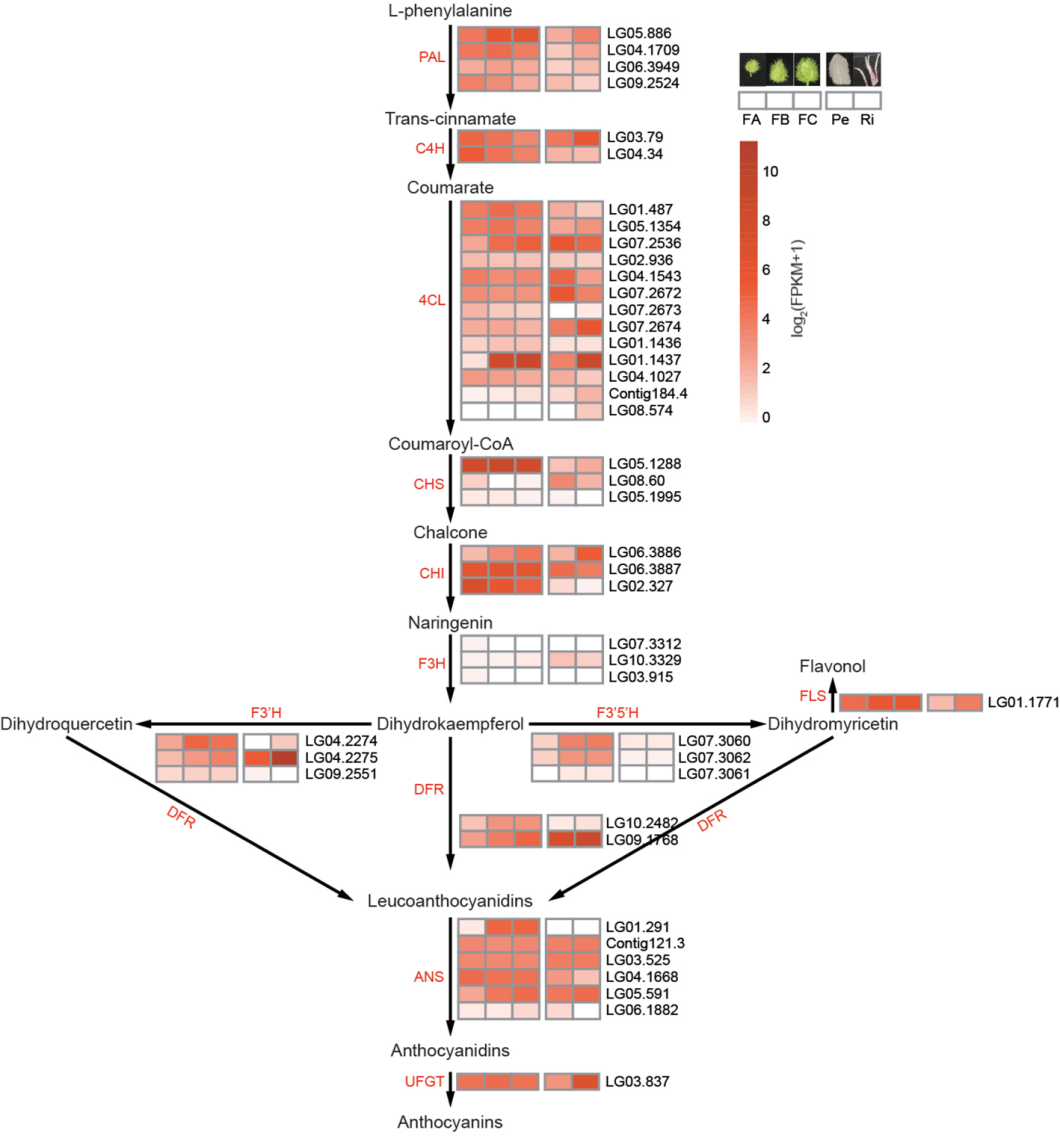
The expression levels of anthocyanin biosynthetic genes in *P. foetida*. Heatmaps show the expression levels of anthocyanin biosynthetic genes in small (FA), intermediate (FB), and large (FC) flower buds, as well as petals (Pe) and radii (Ri) of *P. foetida*. The genes were identified by BLAST searches. Abbreviations: PAL, phenylalanine ammonia-lyase; C4H, cinnamate-4-hydroxylase; 4CL, 4-coumarate CoA ligase; CHS, chalcone synthase; CHI, chalcone isomerase; F3H, flavanone 3-hydroxylase; F3’H, flavanone 3′-hydroxylase; F3’5’H, flavanone 3′,5′-hydroxylase; FLS, flavonol synthase; DFR, dihydroflavonol 4-reductase; ANS, anthocyanidin synthase; and UFGT, UDP-flavonoid glucosyltransferase

Color and scent are important floral traits to attract pollinators (Glover 2011). Various pigments, including anthocyanins, betalains, and carotenoids, contribute to flower coloration (Tanaka et al. 2008). The flowers of *P. foetida* exhibit pale purple or pink pigmentation in the basal part of the inner radii, the tips of pali (Pa), and the androgynophore (Ag) (Additional file 1: Fig. S6). We thus examined the expression patterns of the genes involved in anthocyanin biosynthesis using our transcriptomic data (Fig. 6; Additional file 8: Table S21). Our analysis revealed higher expression levels of *flavanone 3′-hydroxylase* (*F3’H*; LG04.2274, LG04.2275), *anthocyanidin synthase* (*ANS*; LG05.591), and *UDP-flavonoid glucosyltransferase* (*UFGT*; LG03.837) genes in the pigmented radii (Ri) compared to the white petals (Pe; Fig. 6). We also observed that genes such as *flavanone 3′-hydroxylase* (*F3’H*; LG04.2274, LG04.2275), *flavanone 3′,5′-hydroxylase* (*F3’5’H*; LG07.3060, LG07.3062), *dihydroflavonol 4-reductase* (*DFR*; LG10.2482, LG09.1768), and *anthocyanidin synthase* (*ANS*; LG01.291, LG05.591) were expressed at lower levels in small flower buds (FA) compared to medium (FB) and large (FC) flower buds (Additional file 1: Fig. S6g-i), which suggests that the accumulation of anthocyanin likely occurred relatively late in flower development and that these genes may be responsible for the coloration of radii, pali, and the androgynophore of the flower in *P. foetida*.

**Fig. 7:**
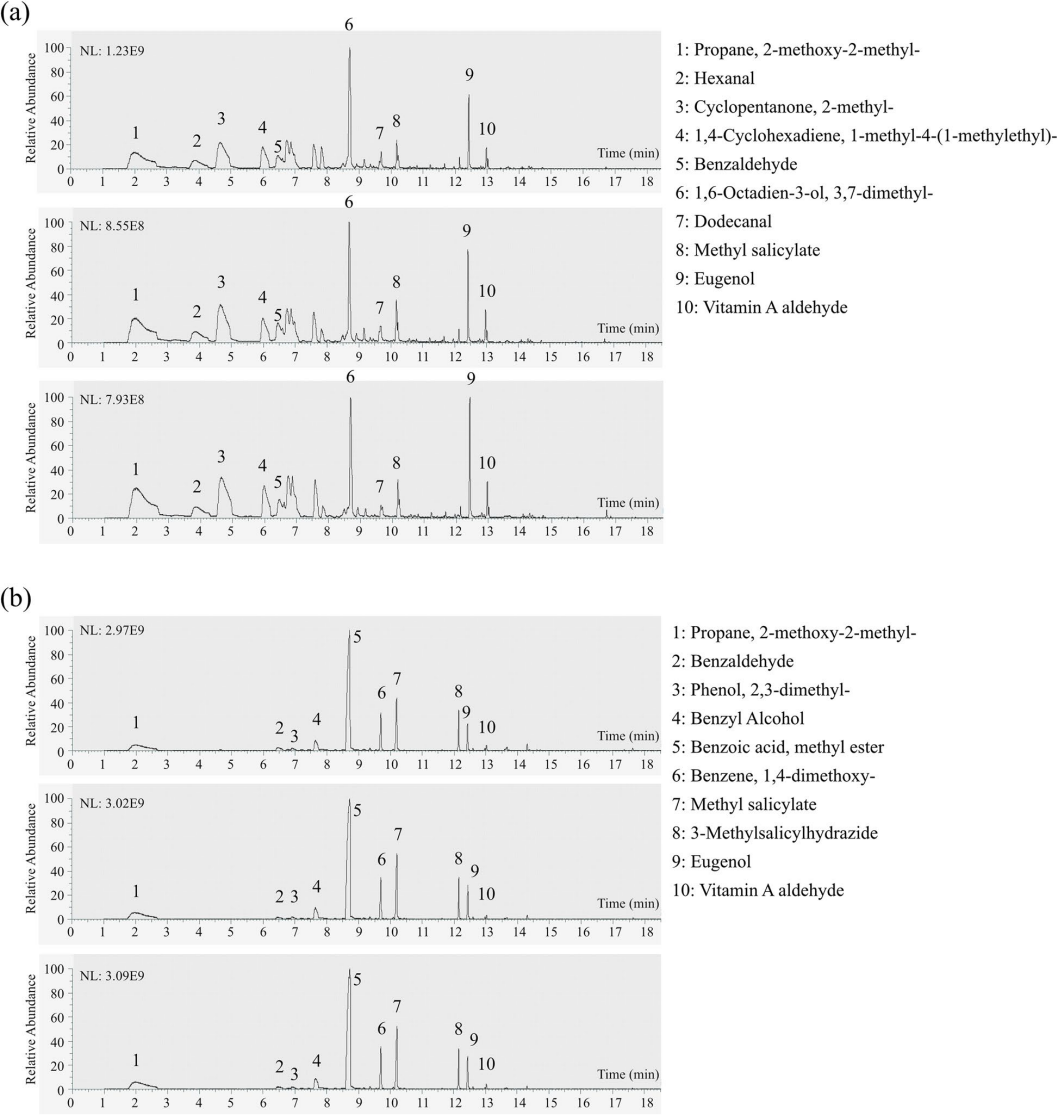
Volatile compounds in *P. foetida* flowers. **a**, **b** GC peaks for three replicates of (**a**) bracts and (**b**) the remaining parts of *P. foetida* flowers. Volatile compounds were identified by GC-MS. The peaks were compared against NIST library using Xcalibur software. Names of compounds (black integer labels in the graphs) were shown on the right

Floral volatile organic compounds (VOCs) are a mixture of plant metabolites with multiple functions (Dotterl and Gershenzon 2023). To understand the chemical compositions of floral scent in *P. foetida*, we performed Gas Chromatography-Mass Spectrometry (GC-MS) analysis for bracts and the fully open flowers separately. We found that the bracts and the fully opened flowers of *P. foetida* were composed of both major and minor VOCs, in which the major shared VOCs were 2-methoxy-2methyl-propane, benzaldehyde, methyl salicylate, eugenol, and vitamin A aldehyde (Additional file 1: Fig. S5). The main unique compound detected in the flowers (without bracts) was methyl 2-methoxy benzoate (or benzoic acid, methyl ester; Fig. 7b). According to our transcriptomic data, the expression levels of the benzoic acid methyl transferases (BSMTs) genes were high in the radii (LG01.282) and bracts (LG01.700; Additional file 1: Fig. S11a; Additional file 8: Table S22). Moreover, the main unique compound detected in the bracts was linalool (or 1,6-Octadien-3-ol, 3,7-dimethy1-; Fig. 7a). Consistently, we found that the expression level of linalool synthase (LG07.309) was the highest in the bracts followed by sepals (Additional file 5: Table S11). According to the literature, the functions of these floral VOCs are under the selection of both pollinators and florivores/herbivores, and often co-evolve with the color and morphology of flowers (Schiestl 2015).

## Discussion

In this work, we present a chromosome-scale genome assembly of *P. foetida*. Through comparing repeat content with the close relative *P. edulis* and analyzing transcriptomic data of different floral organs and developmental stages, we broadly characterized the genomic underpinnings of genome size variation and floral traits in *Passiflora* species, establishing a solid foundation for future research endeavors and flower breeding programs.

### The activity of transposable elements contributes to genome size variation between two *Passiflora* species

Genome sizes vary more than ten-fold between some *Passiflora* species. According to a previous study (Yotoko et al. 2011), the genome size of *P. foetida* is inferred to be smaller than that of the common ancestor between *P. foetida* and *P. edulis* while *P. edulis* is inferred to be larger, however what genomic changes were responsible for these differences in size was unknown. Genome size differences have been previously studied in *P. organensis* but the genome was only a draft version and this species was from the *Decaloba* subgenus (Costa et al. 2021). In the present study, we compared WGD/WGT events and TE contents based on chromosome-level genomes of *P. foetida* and *P. edulis.* We found no independent WGD events following the divergence of these two species suggesting that the disparity in genome sizes was not caused by polyploidization but was primarily driven by differences in TE activity, particularly the Gypsy superfamily of LTR-RTs (Fig. 3). After further characterization of LTR-RT lineages, we concluded that expansion of three LTR-RT lineages (Angela, Athila, Tekay) in *P. edulis* and more rapid elimination of two of them (Angela, Athila) in *P. foetida* were the main drivers of differences in genome size. Interestingly Angela, Athila, and Tekay LTR-RT lineages are all known for being especially long in LTR length, which may be associated with higher activity of homologous recombination activity (Neumann et al. 2019). In the grass species *Brachypodium distachyon*, the Angela lineage of LTR-RT was identified as the most active, with high copy number and high-turnover rates (Stritt et al. 2020).

An interesting question is what mechanisms underlie this differential removal of LTRs in *P. foetida* compared with *P. edulis*. A previous study in cotton (*Gossypium*) suggested that genome expansion mediated by episodic recent LTR-RT amplification was readily reversed by higher rate of DNA loss in smaller genomes (Hawkins et al. 2009), which was perhaps controlled by the intrinsic dynamics of TE proliferation and removal (Novak et al. 2020). Besides genomic factors, ecological factors such as generation time, population size, and nutrients were also proposed to affect the evolution of genome sizes (Leitch and Leitch 2012). In the *Passiflora* genus, genome size has been shown to correlate with cytological and phenotypical traits, such as flower size (Yotoko et al. 2011; Bugallo et al. 2023). Thus, these traits could be further under the selection by pollinators or by humans for ornamental values. As with having carnivorous bracts, *P. foetida* can live better in nutrient-deficient habitats, compared with other *Passiflora* species with larger genome size, because it needs fewer resources to build its smaller genome and smaller reproductive organs. Whether LTR-RT activity is the general mechanism of genome size variation, and whether the same LTR-RT lineages are involved in most cases of genome expansion in *Passiflora* will require the generation of more chromosome-level genome assemblies across the genus.

In addition to expanded genome size, TE insertions can play a crucial role in driving rapid phenotypic variation in plants. For instance, in natural populations of *Capsella rubella*, TE insertions have been shown to correlate with changes in flowering time by affecting the expression levels of the *FLOWERING LOCUS C (FLC)* gene (Niu et al. 2019). TE insertion polymorphisms (TIPs) were shown to be crucial in the domestication of *Brassica rapa* through modifying gene expression levels and altering gene structures (Cai et al. 2022). In the domestication of rice (*Oryza sativa*), TIPs were also frequently associated with changes in regulatory genes (Castanera et al. 2023). Thus, the dynamic patterns of TE distribution in the promoter regions of ABCE floral genes might be an important contributor to modifying the gene expression patterns (Fig. 5c), which could potentially result in different floral traits in *Passiflora* species.

### MADS-box genes are important in the specialized floral development of *P. foetida*

Data from differential expression and copy number variation of MIKC-type MADS-box genes may challenge the classical ABCE model of floral organ development (Teo et al. 2019). For example, in *Bougainvillea glabra*, the expression of A- and B-class genes in bracts were associated with the large size and brilliant color of these sub-floral organs (Zhang et al. 2022b). In orchids, B-class and *AGAMOUS-like 6* (*AGL6*) genes evolved additional functions, including regulating anthocyanin accumulation and red spot formation in the flowers (Hsu et al. 2021). Before chromosome-level genomes were available, researchers studied the ontogeny of the unique flower features in *Passiflora* species based on quantitative Real-Time Reverse Transcription PCR (qRT-PCR) and in situ hybridization (Costa et al. 2019; Pamponet et al. 2019). These studies showed that A-class genes (*AP1* and *FRUITFULL* [*FUL*]) in *P. edulis* were highly expressed in many floral organs, including bracts and tendrils (Scorza et al. 2017). Additional comparative studies in multiple *Passiflora* species also indicated that the *AP1* gene might play important roles in regulating bract and tendril development (Hernandes-Lopes et al. 2019).

In the classical ABC model, expression of only A-class genes specifies sepals, expression of both A- and B-class genes specifies petals, expression of both B- and C-class genes specifies stamens, expression of only C-class genes specifies carpels (Coen and Meyerowitz 1991). In our analyses, the combination of phylogenetic and transcriptomic data showed that these genes were expressed in multiple whorls, including the extra whorls between the petals and stamens. It appeared that some copies of the floral ABC genes still exhibited classical expression patterns in the four whorls of sepals, petals, stamens, and carpels (Fig. 5b). All three A-class genes were expressed in the glandular bracts, while only one copy showed high expression levels in the sepals, petals and additional whorls. This normally very tiny floral organ was not included in the classic ABC model, but could also have ornamental values in some species, such as *Curcuma alismatifolia* (Liao et al. 2022). According to the expression patterns, the B-class gene LG08.1025 appeared to act as a B-function gene, because it was expressed at very low levels in sepals and pistils, but highly expressed in petals and stamens (Fig. 5). However other B-class-like genes, LG01.683 in particular, were expressed in sepals and pistils at high levels. The four C-class genes were assigned to two groups based on phylogenetic relationships and similar expression patterns. Thus, expansion of B- and C-class genes and their combined dosage might be one of the factors underlying the unique floral features in *Passiflora* species. Moreover, we also compared the chromosomal locations and flanking sequences around A-class genes in *P. foetida* and *P. edulis*. The results from these analyses indicated that these genes were being rapidly translocated potentially because of TE-mediated recombination processes and that TEs were more abundant in the promotors of A-class genes in *P. edulis* (Additional file 1: Fig. S5). This additional genome plasticity might contribute to the diversification of flower morphology and pollination strategies of *Passiflora* species. With chromosome-level genome assemblies, we were able to study MADS-box genes in more detail, which has helped to improve our understanding of the development of the distinctive *Passiflora* flowers and will enhance the ornamental value through marker assisted breeding.

### Floral color and scent in *P. foetida*

Recently, it was shown that the flowers of *P. foetida* exhibited both self-pollination and cross-pollination, with the intermediate curvature of stigma facilitating the pollinators (Mallikarjuna 2023). As suggested by our transcriptomic data, the biosynthesis and accumulation of anthocyanin occurred in late stages of floral development. The production of the visual signal (anthocyanin) only when needed saves energy and resources for *P. foetida*, which in addition could be learned and selected by pollinators (mutualists).

Besides, the formation of floral scent or VOCs is in conflict selection by both pollinators and florivores/herbivores (Schiestl 2015). The methyl benzoate emitted by the fully open flowers of *P. foetida* can be a signal to attract pollinators such as bees (Additional file 1: Fig. S6c), while remain highly toxic towards certain pests (Zhao et al. 2022). Interestingly, in *Petunia* RNAi lines that cannot produce methyl benzoate, damage rates were dramatically reduced compared with normal flowers, which emitted very similar VOCs as *P. foetida* (Kessler et al. 2013). Generally, whether it is beneficial or harmful to the plants depends on the relative abundance of pollinators and florivores/herbivores in the environment and the strength of VOC emission (Schiestl 2015). The glandular bracts of *P. foetida* emitted a major scent component linalool (Additional file 1: Fig. S11a). This common VOC can function in both floral defense and pollinator attraction depending on the ratio of the two enantiomers, which in turn is determined by the spatiotemporal expression of biosynthetic genes (Raguso 2016). Perhaps in *P. foetida*, linalool emitted by the bracts was more a defensive compound, which required to be verified by advanced metabolomics technologies (Yan et al. 2022). Further studies are needed to completely understand the ecological roles of floral scent in *P. foetida*.

## Conclusion

In conclusion, our newly assembled genome and the transcriptomic data of *P. foetida* provide a valuable new resource for comprehending the important roles played by LTR-RT lineages in genome size variation. Furthermore, our findings shed light on the potential roles of expression and copy number variation of MADS-box genes to flower development in *Passiflora* species.

## Methods

### Plant materials

Branch tips, including young leaves and tendrils of *P. foetida*, were collected from one plant near Luogu Mountain Park (114.4926° E, 22.6007° N) at Dapeng New District, Shenzhen, Guangdong province, China for Illumina, Oxford Nanopore Technologies (ONT), and Hi-C sequencing. The leaves, stems, tendrils, young fruit, and flower buds were used for RNA-seq and were collected from multiple plants. A total of 172 flowers were collected from one plant and kept frozen until the different flower parts could be dissected. The samples of floral organs were ground thoroughly in liquid nitrogen for RNA extraction and RNA sequencing.

### DNA extraction, library construction and sequencing

Genomic DNA was prepared by the sodium dodecyl sulfate (SDS) method followed by purification with QIAGEN® Genomic DNA extraction kit (Cat#13343, QIAGEN). Purified DNA was quantified using Nanodrop (ND-1000, Thermo Scientific, Wilmington, DE, USA) and Qubit® 3.0 Fluorometers (Invitrogen, USA). For Illumina sequencing, libraries were prepared with fragmented DNA (200–400 bp) through end-repair, 3′ adenylated, adapter-ligation, PCR amplification with the products recovered by the AxyPrep Mag PCR clean up Kit. The double stranded PCR products were heat denatured and circularized by the splint oligo sequence and sequenced on an MGISEQ instrument. For ONT sequencing, additional steps after DNA extraction included selecting long DNA fragments using the BluePippin system (Sage Science, USA), A-ligation repair of DNA ends using NEBNext Ultra II End Repair/dA-tailing Kit (Cat# E7546), and adapter ligation using the LSK109 kit. The resulting ONT library were sequenced on a Nanopore PromethION sequencer (Oxford Nanopore Technologies, UK) instrument at Nextomics. To construct the Hi-C library for sequencing on an MGI-2000 platform, freshly collect tissues were first fixed using 2% formaldehyde (glycine was used to stop crosslinking) and ground into powder and resuspended to isolate the nuclei. Then, DpnII, biotin-14dCTP, and T4 DNA polymerase were sequentially added. The ligated DNA was sheared into 300–600 bp fragments, and then was blunt-end repaired and A-tailed, followed by purification through biotin-streptavidin-mediated pull down.

### Survey of genome size and heterozygosity

To estimate the genome size and heterozygosity, 23.71 Gb of clean Illumina paired-ended reads were analyzed using Kmerfreq (17-mer) in gse (v1.0.2) software.

### Genome assembly and assessment

A total of 62.15 Gb of Nanopore single-pass reads were assembled with NextDenovo (v2.3.1; Hu et al. 2023) to get a preliminary assembly of 424.85 Mb (contig N50 = 12.92 Mb). This preliminary assembly was corrected using the Nanopore reads, and clean Illumina reads using Nextpolish (v1.3.0; Hu et al. 2020). After removing organellar contigs, we used Hi-C sequencing data (87.50 Gb clean Illumina paired-ended reads) to anchor the contigs using LACHESIS (https://github.com/shendurelab /LACHESIS: commit 2e27abb; Burton et al. 2013).

Merqury (version 1.3; Rhie et al. 2020) was used to assess the correctness (QV) and completeness of the final genome assembly. Potential contamination sequences were estimated using Blobtools (version 1.1.1; Laetsch and Blaxter 2017). Potential redundant sequences were evaluated using purge_haplotigs (version 1.1.2; Roach et al. 2018).

### Genome annotation and evaluation

TEs in the assembly were first identified using EDTA (v1.9; Ou et al. 2019). The identified LTR-RTs were further classified into individual clades using TEsorter (v1.4.6; Zhang et al. 2022a) against the REXdb database (Neumann et al. 2019). Protein-coding genes were predicted using homology-based, transcript and ab initio evidence. First, the peptides from *Passiflora edulis* (CNA0017758), *Populus trichocarpa* (GCF_000002775.4) and *Hevea brasiliensis* (GCF_001654055.1) were aligned to the repeat-masked assembly using Exonerate (v2.2.0 R11; Slater and Birney 2005). The RNA-seq data of different samples was aligned to the reference by HISAT2 (v2.1.0; Kim et al. 2019), assembled by StringTie (v2.1.4; Pertea et al. 2015) and predicted by TransDecoder (v5.5.0; Haas et al. 2013). De novo transcript assembly of the two samples were performed by Trinity (v2.14.0; Grabherr et al. 2011). Both de novo and reference-based transcript predictions were used to generate a database using PASA (v2.5.2; Haas et al. 2003). For *ab inito* prediction by AUGUSTUS (v3.4.0 F2; Stanke et al. 2006), both homology-based and transcript-based evidence were used to train the model. Finally, all predictions were merged using EVidenceModeler (EVM, v1.1.1; Haas et al. 2008). A total of 30,617 protein models were predicted with a BUSCO value of 94.2%. Annotation of protein functions was performed using BLASTP by searching against SwissProt, GO, KEGG, Pfam (Finn et al. 2014) and NR databases. Annotation of non-coding RNAs were performed using Rfam 14 (Griffiths-Jones et al. 2003), tRNAscanSE (v2.0; Lowe and Eddy 1997) and Infernal (v1.1.3; Nawrocki and Eddy 2013).

### Genome evolution analysis

Orthologous gene families among *P. foetida* and nine other species were constructed using OrthoFinder (v2.5.2; Emms and Kelly, 2019). A phylogenetic tree was constructed using RAxML (v8.2.12; Stamatakis 2014) based on the first and second codon sites in 278 single-copy gene families. The divergence times in the phylogenetic tree were estimated using treePL (v1.0; Smith and O'Meara 2012) with calibrations taken from the TimeTree database (http://www.timetree.org/). CAFÉ (v4.1; De Bie et al. 2006) was used to identify expanded and contracted gene families. Synteny of genes were analyzed using MCScanX (v0.8; Wang et al. 2012) and visualized using the python module JCVI (v1.1.14; Tang et al. 2008).

### Whole-genome duplication analysis

The synonymous substitution rate (*K*_*S*_) of homologous genes in MCScanX-identified syntenic blocks and was calculated to determine recent WGD or WGT events. Dot-plots were generated to further assess WGD events.

### Estimation of LTR insertion time

Solo and intact LTR TEs were calculated by the scripts “solo_finder.pl” and “intact_finder_coarse.pl” in LTR_retriever (Ou and Jiang 2018). The solo/intact ratios were calculated by “solo_intact_ratio.pl”. LTR insertion time was also calculated by LTR_retriever, where μ = 5.35e-9 (using estimated absolute rates of silent-site divergence from De La Torre et al. 2017).

### RNA extraction, cDNA preparation, sequencing and analysis

Total RNA from each sample was extracted using the RNAprep pure Plant Kit (TIANGEN) and was purified using poly-T beads. After synthesis, cDNA was first end-repaired, A-tailed, adapter-ligated, PCR-amplified, denatured, and circularized. The resulting products were quality-controlled using gel electrophoresis, Nanodrop (ND-1000, Thermo Scientific, Wilmington, DE, USA), and an Agilent 2100 Bioanalyzer System. RNA sequencing was performed using the MGIseq2000 platform. Raw reads were first quality controlled using SOAPnuke (v2.1.0; Chen et al. 2018) and further filtered using Trimmomatic (v0.39; Bolger et al. 2014). The resulting clean reads were aligned against the *P. foetida* genome assembly. StringTie (v2.1.4; Pertea et al. 2015) was used to get the Fragments per Kilobase Million (FPKM) value for each transcript. PCA plots, correlation heatmap, and the correlation coefficient matrix was generated using the R package ggplot2. DEGs between sample pairs were analyzed using the R package DESeq2. KEGG and GO enrichment analysis was performed using the R packages KEGGREST (v1.30.1) and AnnotationForge (v1.32.0), respectively.

### Identification and analysis of MADS-box and floral metabolite genes

First, candidate MADS-box genes from *P. foetida* and *P. edulis* were identified by hmmsearch (v3.3.2; cutoff e-value < 0.1) using downloaded hidden Markov model (HMM) profiles of SRF-type-I (PF00319) and MEF2-type-II (PF09047) as queries, combining the BLASTp (cutoff e-value < 0.0001) results using *Arabidopsis* homologs as queries (Parenicova et al. 2003). Then these candidate proteins were searched for conserved domains in the Pfam database. MIKC-type MADS-box genes with a K-domain were aligned by MAFFT (v7.490; Katoh and Standley 2013) and were used to build a maximum likelihood tree using IQTree2 (v2.0.7; Minh et al. 2020). TE contents surrounding these MADS-box genes were analyzed using MCScanX and visualized using LINKVIEW2 (v1.0.5).

Genes involved in the biosynthesis of anthocyanin and methyl benzoate were identified by BLASTP, in which the azalea (*Rhododendron simsii*) homologs were chosen as queries because of the relatively complete annotation of these genes in this species. All search results were compared to the results of genome annotation.

### Metabolomic analysis of volatile compounds

Volatile compounds were collected by solid-phase microextraction. Samples from the bracts (Br) and the remaining parts of six fully open flowers of *P. foetida* were collected and cut to fit into 15 ml headspace bottles with three replicates. These collected samples were settled in the bottle for 2 hours, inserted with the extraction fiber for 0.5 hour respectively, and then inserted into the GC injection port for 3 min at 250 °C. The extracted compounds were measured using a GCMS-QP2010 system (Shimadzu, Japan), equipped with a DB-5MS column (30 m × 0.25 mm, 0.25 ms). He (99.999%) was used as carrier gas (split injection mode, 27.2 mL/min total flow rate). Ion source and interface temperatures were 200 °C and 250 °C, respectively. The detailed GC-MS run parameters used in the current study are described previously in detail (Zhang et al. 2022c), i.e., 1 KV detector voltage and 30 to 500 *m/z* full scanning mass range, 40 min total heating time (50 °C, 1 min; increasing 5 °C per minute; 250 °C, 2 min). The identities of major volatile compounds based on the chromatograms and mass spectrograms were compared with the NIST2011 library and were checked against the Pherobase database, the hit with maximal probability was kept for each peak (http://www.pherobase.com).

### Supplementary Information

**Additional file 1:** **Fig. S1.** Survey of *P. foetida* genome using kmerfreq and assessment of the final assembly. (a) Density plot shows 17-mer distribution of 25.70 Gb of short reads, indicating a genome size of 456.46 Mb with high repeat content and low heterozygosity. (b) Plots indicates very low contamination using Blobtools. (c) Histogram indicates very few redundant sequences using purge_haplotigs. **Fig. S2.** Hi-C contact map of the *P. foetida* genome assembly. Heatmap shows 92.18% of the assembled contigs were anchored into ten pseudochromosomes (LG01 – LG10) with LACHESIS. **Fig. S3.** GO/KEGG enrichment of expanded and contracted gene families in *P. foetida*. (a, b) GO enrichment analysis of (a) expanded and (b) contracted gene families in *P. foetida*. (c, d) KEGG enrichment analysis of (c) expanded and (d) contracted gene families in *P. foetida*. **Fig. S4.** One shared recent WGD event between *P. foetida* and *P. edulis*. (a) A *Ks* density plot shows one recent WGD event shared by *P. foetida* and *P. edulis*. (b, c) Dot plots show that this recent WGD event was shared by *P. foetida* and *P. edulis*. Colors indicate *Ks* values (sidebar). **Fig. S5.** TE contents surrounding A-class MADS-box genes in *P. foetida* and *P. edulis*. (a) TE contents with ten genes both upstream and downstream of A-class MADS-box genes are shown. (b) A microsyntenic block from LG10.196 to LG10.214 (containing LG10.207/AP1 in *P. foetida*) was shown. **Fig. S6.** Samples of *P. foetida* used for RNA sequencing. (a, b) Photos show a segment of stem of *P. foetida* in the morning of two successive days. (c) A fully opened flower attracted a honeybee pollinator. (d, e) Photos show (d) dissected parts of floral organs, (e) the stem including a leaf and a tendril (see also panel f). (g-j) Photos show flower buds of different sizes and (j) young fruits, which were all surrounded by persistent glandular bracts. **Fig. S7.** The numbers of SHEGs in different samples. Heatmap shows the numbers of specifically highly expressed genes (SHEGs) for each sample group. Values are the numbers of upregulated DEGs of one sample (vertical) versus another (horizontal). Abbreviations: Br, bracts; Se, sepals; Pe, petals; Ri, radii; Pa, pali; Ag, androgynophore; St, stamens; and Pi, pistils. **Fig. S8.** GO enrichment of SHEGs in different samples. Abbreviations of GO function classifications: BP, biological process; CC: cellular component; MF, molecular function. The x-axis indicates gene ratio. **Fig. S9.** KEGG enrichment of SHEGs in different samples. The x-axis in each subfigure indicates the number of enriched DEGs. **Fig. S10.** GO/KEGG enrichment of upregulated DEGs in Pi and St. (a) GO and (b) KEGG enrichment analysis of pistils (Pi). (c) GO and (d) KEGG enrichment analysis of stamens (St). The x-axes in panels b and d indicate the number of enriched DEGs. **Fig. S11.** The expression levels of biosynthetic genes of major floral volatiles in *P. foetida*. Heatmaps show the expression levels of methyl benzoate biosynthetic genes in different floral whorls of *P. foetida*. The genes were identified by BLAST searches. Abbreviations in a: PAL, phenylalanine ammonia-lyase; PXA1, peroxisomal ATP-binding cassette transporter 1; CNL, cinnamoyl-CoA ligase; CHD, cinnamoyl-CoA hydratase/dehydrogenase; KAT, 3-ketoacyl CoA thiolase; TE, thioesterase; BSMT, benzoic acid/salicylic acid carboxyl methyltransferases; and BALD, benzaldehyde dehydrogenase.

**Additional file 2:** **Table S1.** Statistics of whole genome sequencing data. **Table S2.** Table S2 Statistics of Hi-C scaffolded genome assembly of *P. foetida*. **Table S3.** Statistics of proteins with function annotations. **Table S4.** Statistics of non-coding RNA annotations.

**Additional file 3:** **Table S5.** GO enrichment analysis of expanded and contracted gene families in *P. foetida.* **Table S6.** KEGG enrichment analysis of expanded and contracted gene families in *P. foetida*.

**Additional file 4:** **Table S7.** Statistics of repeat annotation by EDTA. **Table S8.** Statistics of clade-level classification of LTR-RTs by TEsorter. **Table S9.** Statistics of clade-level classification of intact LTR-RTs by TEsorter. **Table S10.** Ratios of solo/intact LTR-RT clades.

**Additional file 5:** **Table S11.** FPKM values of all samples for RNA-seq.

**Additional file 6:** **Table S12**. Differentially expressed gene between sample pairs.

**Additional file 7:** **Table S13.** Specifically highly expressed genes in different samples. **Table S14.** GO enrichment analysis of SHEGs in different samples. **Table S15.** KEGG enrichment analysis of SHEGs in different samples. **Table S16.** GO enrichment analysis of up-regulated DEGs in Pi. **Table S17.** KEGG enrichment analysis of up-regulated DEGs in Pi. **Table S18.** GO enrichment analysis of up-regulated DEGs in St. **Table S19.** KEGG enrichment analysis of up-regulated DEGs in St. **Table S20.** The expression levels of Type-II MADS-box genes in floral organs of *P. foetida*.

**Additional file 8:** **Table S21.** Expression levels of gene in anthocyanin biosynthesis. **Table S22.** Expression levels of gene in benzoate biosynthesis.

## Data Availability

The original contributions presented in the study are publicly available. The raw sequence data and the genome assembly with annotation reported in this paper have been deposited in the Genome Sequence Archive in the National Genomics Data Center, China National Center for Bioinformation / Beijing Institute of Genomics, Chinese Academy of Sciences (PRJCA020083), which is publicly accessible at https://ngdc.cncb.ac.cn.

## Abbreviations

*AGL6*: *AGAMOUS-like 6*
*AMT1*: *AMMONIUM TRANSPORTER 1*
*AP2*: *APETALA2*
BALD: benzaldehyde dehydrogenase
BSMT: benzoic acid/salicylic acid carboxyl methyltransferase
BUSCO: Benchmarking Universal Single-Copy Orthologs
CHD: cinnamoyl-CoA hydratase/dehydrogenase
CNL: cinnamoyl-CoA ligase
DEG: differentially expressed gene
*FLC*: *FLOWERING LOCUS C*
FPKM: the Fragments per Kilobase Million
*FUL*: *FRUITFULL*
GC-MS: Gas Chromatography-Mass Spectrometry
GO: Gene Ontology
HMM: hidden Markov model
KAT: 3-ketoacyl CoA thiolase
KEGG: Kyoto Encyclopedia of Genes and Genomes
LAI: LTR assembly index
LTR-RT: long terminal repeat retrotransposon
ONT: Oxford Nanopore Technologies
PAL: phenylalanine ammonia-lyase
PCA: principal component analysis
*PI*: *PISTILLATA*
PXA1: peroxisomal ATP-binding cassette transporter 1
qRT-PCR: quantitative Real-Time Reverse Transcription PCR
SDS: sodium dodecyl sulfate
SHEG: specifically highly expressed gene
TE: transposable element
TE: thioesterase
TIP: TE insertion polymorphism
VOC: volatile organic compound
WGD: whole genome duplication
WGT: whole genome triplication
*WRI1*: *WRINKLED1*

## References

[CR1] Baum DA, Whitlock BA (1999). Plant development: genetic clues to petal evolution. Curr Biol.

[CR2] Bolger AM, Lohse M, Usadel B (2014). Trimmomatic: a flexible trimmer for Illumina sequence data. Bioinformatics.

[CR3] Bugallo VL, Facciuto GR, Poggio L. Genome size in Argentinean species of *Passiflora* genus: cytological and phenotypical correlates. Rodriguésia. 2023;74

[CR4] Burton JN, Adey A, Patwardhan RP, Qiu R, Kitzman JO, Shendure J (2013). Chromosome-scale scaffolding of *de novo* genome assemblies based on chromatin interactions. Nat Biotechnol.

[CR5] Cai X, Lin R, Liang J, King GJ, Wu J, Wang X (2022). Transposable element insertion: a hidden major source of domesticated phenotypic variation in *Brassica rapa*. Plant Biotechnol J.

[CR6] Castanera R, Morales-Diaz N, Gupta S, Purugganan M, Casacuberta JM. Transposons are important contributors to gene expression variability under selection in rice populations. Elife. 2023;12 10.7554/eLife.86324 PMC10393045 37467142

[CR7] Cerqueira-Silva CB, Jesus ON, Santos ES, Correa RX, Souza AP (2014). Genetic breeding and diversity of the genus *Passiflora*: progress and perspectives in molecular and genetic studies. Int J Mol Sci.

[CR8] Chen Y, Chen Y, Shi C, Huang Z, Zhang Y, Li S, Li Y, Ye J, Yu C, Li Z, Zhang X, Wang J, Yang H, Fang L, Chen Q (2018). SOAPnuke: a MapReduce acceleration-supported software for integrated quality control and preprocessing of high-throughput sequencing data. Gigascience.

[CR9] Classen-Bockhoff R, Meyer C (2016). Space matters: meristem expansion triggers corona formation in *Passiflora*. Ann Bot.

[CR10] Coen ES, Meyerowitz EM (1991). The war of the whorls: genetic interactions controlling flower development. Nature.

[CR11] Costa ZP, Cauz-Santos LA, Ragagnin GT, Van Sluys MA, Dornelas MC, Berges H, de Mello Varani A, Vieira MLC (2019). Transposable element discovery and characterization of LTR-retrotransposon evolutionary lineages in the tropical fruit species *Passiflora edulis*. Mol Biol Rep.

[CR12] Costa ZP, Varani AM, Cauz-Santos LA, Sader MA, Giopatto HA, Zirpoli B, Callot C, Cauet S, Marande W, Souza Cardoso JL, Pinheiro DG, Kitajima JP, Dornelas MC, Harand AP, Berges H, Monteiro-Vitorello CB, Carneiro Vieira ML (2021). A genome sequence resource for the genus *Passiflora*, the genome of the wild diploid species *Passiflora organensis*. Plant Genome.

[CR13] De Bie T, Cristianini N, Demuth JP, Hahn MW (2006). CAFE: a computational tool for the study of gene family evolution. Bioinformatics.

[CR14] De La Torre AR, Li Z, Van de Peer Y, Ingvarsson PK (2017). Contrasting rates of molecular evolution and patterns of selection among gymnosperms and flowering plants. Mol Biol Evol.

[CR15] de Oliveira GA, de Castilhos F, Renard CM-GC, Bureau S (2014). Comparison of NIR and MIR spectroscopic methods for determination of individual sugars, organic acids and carotenoids in passion fruit. Food Res Int.

[CR16] Di Cristina M, Sessa G, Dolan L, Linstead P, Baima S, Ruberti I, Morelli G (1996). The *Arabidopsis* Athb-10 (GLABRA2) is an HD-zip protein required for regulation of root hair development. Plant J.

[CR17] Dotterl S, Gershenzon J. Chemistry, biosynthesis and biology of floral volatiles: roles in pollination and other functions. Nat Prod Rep. 2023; 10.1039/d3np00024a 37661854

[CR18] Emms DM, Kelly S. OrthoFinder: phylogenetic orthology inference for comparative genomics. Genome Biol. 2019;20(1):238. 10.1186/s13059-019-1832-y. 10.1186/s13059-019-1832-y PMC6857279 31727128

[CR19] Finn RD, Bateman A, Clements J, Coggill P, Eberhardt RY, Eddy SR, Heger A, Hetherington K, Holm L, Mistry J, Sonnhammer EL, Tate J, Punta M (2014). Pfam: the protein families database. Nucleic Acids Res.

[CR20] Fonseca AMA, Geraldi MV, Junior MRM, Silvestre AJD, Rocha SM (2022). Purple passion fruit (*Passiflora edulis* f. edulis): a comprehensive review on the nutritional value, phytochemical profile and associated health effects. Food Res Int.

[CR21] Fukushima K, Fang X, Alvarez-Ponce D, Cai H, Carretero-Paulet L, Chen C, Chang TH, Farr KM, Fujita T, Hiwatashi Y, Hoshi Y, Imai T, Kasahara M, Librado P, Mao L, Mori H, Nishiyama T, Nozawa M, Palfalvi G, Hasebe M (2017). Genome of the pitcher plant *Cephalotus* reveals genetic changes associated with carnivory. Nat Ecol Evol.

[CR22] Glover BJ (2011). Pollinator attraction: the importance of looking good and smelling nice. Curr Biol.

[CR23] Grabherr MG, Haas BJ, Yassour M, Levin JZ, Thompson DA, Amit I, Adiconis X, Fan L, Raychowdhury R, Zeng Q, Chen Z, Mauceli E, Hacohen N, Gnirke A, Rhind N, di Palma F, Birren BW, Nusbaum C, Lindblad-Toh K, Regev A (2011). Full-length transcriptome assembly from RNA-Seq data without a reference genome. Nat Biotechnol.

[CR24] Griffiths-Jones S, Bateman A, Marshall M, Khanna A, Eddy SR (2003). Rfam: an RNA family database. Nucleic Acids Res.

[CR25] Haas BJ, Delcher AL, Mount SM, Wortman JR, Smith RK, Hannick LI, Maiti R, Ronning CM, Rusch DB, Town CD, Salzberg SL, White O (2003). Improving the *Arabidopsis* genome annotation using maximal transcript alignment assemblies. Nucleic Acids Res.

[CR26] Haas BJ, Papanicolaou A, Yassour M, Grabherr M, Blood PD, Bowden J, Couger MB, Eccles D, Li B, Lieber M, MacManes MD, Ott M, Orvis J, Pochet N, Strozzi F, Weeks N, Westerman R, William T, Dewey CN, Regev A (2013). De novo transcript sequence reconstruction from RNA-seq using the trinity platform for reference generation and analysis. Nat Protoc.

[CR27] Haas BJ, Salzberg SL, Zhu W, Pertea M, Allen JE, Orvis J, White O, Buell CR, Wortman JR (2008). Automated eukaryotic gene structure annotation using EVidenceModeler and the program to assemble spliced alignments. Genome Biol.

[CR28] Hawkins JS, Proulx SR, Rapp RA, Wendel JF (2009). Rapid DNA loss as a counterbalance to genome expansion through retrotransposon proliferation in plants. Proc Natl Acad Sci U S A.

[CR29] He X, Luan F, Yang Y, Wang Z, Zhao Z, Fang J, Wang M, Zuo M, Li Y (2020). *Passiflora edulis*: an insight into current researches on phytochemistry and pharmacology. Front Pharmacol.

[CR30] Hemingway CA, Christensen AR, Malcomber ST (2011). B- and C-class gene expression during corona development of the blue passionflower (Passiflora caerulea, Passifloraceae). Am J Bot.

[CR31] Hernandes-Lopes J, Sousa-Baena MS, Lemos RCC, Correa TCS, Van Sluys MA, Melo-de-Pinna GFA (2019). Toward understanding inflorescence development and architecture in *Passiflora*: insights from comparative anatomy and expression of *APETALA1*. Am J Bot.

[CR32] Hsu HF, Chen WH, Shen YH, Hsu WH, Mao WT, Yang CH (2021). Multifunctional evolution of B and *AGL6* MADS box genes in orchids. Nat Commun.

[CR33] Hu J, Chang X, Zhang Y, Yu X, Qin Y, Sun Y, Zhang L (2021). The pineapple MADS-box gene family and the evolution of early monocot flower. Sci Rep.

[CR34] Hu J, Fan J, Sun Z, Liu S (2020). NextPolish: a fast and efficient genome polishing tool for long-read assembly. Bioinformatics.

[CR35] Hu J, Wang Z, Sun Z, Hu B, Ayoola AO, Liang F, et al. An efficient error correction and accurate assembly tool for noisy long reads. bioRxiv. 2023:2023.2003.2009.531669. 10.1186/s13059-024-03252-4 PMC11046930 38671502

[CR36] Katoh K, Standley DM (2013). MAFFT multiple sequence alignment software version 7: improvements in performance and usability. Mol Biol Evol.

[CR37] Kessler D, Diezel C, Clark DG, Colquhoun TA, Baldwin IT (2013). Petunia flowers solve the defence/apparency dilemma of pollinator attraction by deploying complex floral blends. Ecol Lett.

[CR38] Kim D, Paggi JM, Park C, Bennett C, Salzberg SL (2019). Graph-based genome alignment and genotyping with HISAT2 and HISAT-genotype. Nat Biotechnol.

[CR39] Kong Q, Ma W (2018). WRINKLED1 transcription factor: how much do we know about its regulatory mechanism?. Plant Sci.

[CR40] Kumar S, Stecher G, Suleski M, Hedges SB (2017). TimeTree: a resource for timelines, timetrees, and divergence times. Mol Biol Evol.

[CR41] Laetsch D, Blaxter M. BlobTools: interrogation of genome assemblies. F1000Research. 2017;6(1287)

[CR42] Leitch AR, Leitch IJ (2012). Ecological and genetic factors linked to contrasting genome dynamics in seed plants. New Phytol.

[CR43] Liao X, Ye Y, Zhang X, Peng D, Hou M, Fu G, Tan J, Zhao J, Jiang R, Xu Y, Liu J, Yang J, Liu W, Tembrock LR, Zhu G, Wu Z (2022). The genomic and bulked segregant analysis of *Curcuma alismatifolia* revealed its diverse bract pigmentation. aBIOTECH.

[CR44] Lisch D (2013). How important are transposons for plant evolution?. Nat Rev Genet.

[CR45] Lowe TM, Eddy SR (1997). tRNAscan-SE: a program for improved detection of transfer RNA genes in genomic sequence. Nucleic Acids Res.

[CR46] Ma D, Dong S, Zhang S, Wei X, Xie Q, Ding Q, Xia R, Zhang X (2021). Chromosome-level reference genome assembly provides insights into aroma biosynthesis in passion fruit (*Passiflora edulis*). Mol Ecol Resour.

[CR47] Maeo K, Tokuda T, Ayame A, Mitsui N, Kawai T, Tsukagoshi H, Ishiguro S, Nakamura K (2009). An AP2-type transcription factor, WRINKLED1, of *Arabidopsis thaliana* binds to the AW-box sequence conserved among proximal upstream regions of genes involved in fatty acid synthesis. Plant J.

[CR48] Mallikarjuna RM (2023). Pollination ecology, breeding systems and seed dispersal in *Passiflora foetida* L. (Passifloraceae), a perennial herbaceous climber weed in southern parts of Andhra Pradesh, India.: pollination ecology of *Passiflora foetida* L. (Passifloraceae). Biotropia.

[CR49] Minh BQ, Schmidt HA, Chernomor O, Schrempf D, Woodhams MD, von Haeseler A, Lanfear R (2020). IQ-TREE 2: new models and efficient methods for phylogenetic inference in the genomic era. Mol Biol Evol.

[CR50] Muschner VC, Lorenz AP, Cervi AC, Bonatto SL, Souza-Chies TT, Salzano FM, Freitas LB (2003). A first molecular phylogenetic analysis of *Passiflora* (Passifloraceae). Am J Bot.

[CR51] Nawrocki EP, Eddy SR (2013). Infernal 1.1: 100-fold faster RNA homology searches. Bioinformatics.

[CR52] Neumann P, Novak P, Hostakova N, Macas J (2019). Systematic survey of plant LTR-retrotransposons elucidates phylogenetic relationships of their polyprotein domains and provides a reference for element classification. Mob DNA.

[CR53] Ng M, Yanofsky MF (2001). Function and evolution of the plant MADS-box gene family. Nat Rev Genet.

[CR54] Niu XM, Xu YC, Li ZW, Bian YT, Hou XH, Chen JF, Zou YP, Jiang J, Wu Q, Ge S, Balasubramanian S, Guo YL (2019). Transposable elements drive rapid phenotypic variation in *Capsella rubella*. Proc Natl Acad Sci U S A.

[CR55] Novak P, Guignard MS, Neumann P, Kelly LJ, Mlinarec J, Koblizkova A, Dodsworth S, Kovarik A, Pellicer J, Wang W, Macas J, Leitch IJ, Leitch AR (2020). Repeat-sequence turnover shifts fundamentally in species with large genomes. Nat Plants.

[CR56] Ou S, Jiang N (2018). LTR_retriever: a highly accurate and sensitive program for identification of long terminal repeat retrotransposons. Plant Physiol.

[CR57] Ou S, Su W, Liao Y, Chougule K, Agda JRA, Hellinga AJ, Lugo CSB, Elliott TA, Ware D, Peterson T, Jiang N, Hirsch CN, Hufford MB (2019). Benchmarking transposable element annotation methods for creation of a streamlined, comprehensive pipeline. Genome Biol.

[CR58] Pamponet VCC, Souza MM, Silva GS, Micheli F, de Melo CAF, de Oliveira SG, Costa EA, Correa RX (2019). Low coverage sequencing for repetitive DNA analysis in *Passiflora edulis* Sims: citogenomic characterization of transposable elements and satellite DNA. BMC Genomics.

[CR59] Parenicova L, de Folter S, Kieffer M, Horner DS, Favalli C, Busscher J, Cook HE, Ingram RM, Kater MM, Davies B, Angenent GC, Colombo L (2003). Molecular and phylogenetic analyses of the complete MADS-box transcription factor family in *Arabidopsis*: new openings to the MADS world. Plant Cell.

[CR60] Patil AS, Paikrao HM (2012). Bioassay guided phytometabolites extraction for screening of potent antimicrobials in *Passiflora foetida* L. J Appl Pharm Sci.

[CR61] Pertea M, Pertea GM, Antonescu CM, Chang TC, Mendell JT, Salzberg SL (2015). StringTie enables improved reconstruction of a transcriptome from RNA-seq reads. Nat Biotechnol.

[CR62] Radhamani TR, Sudarshana L, Krishnan R (1995). Defense and carnivory: dual role of bracts in *Passiflora foetida*. J Biosci.

[CR63] Raguso RA (2016). More lessons from linalool: insights gained from a ubiquitous floral volatile. Curr Opin Plant Biol.

[CR64] Rerie WG, Feldmann KA, Marks MD (1994). The GLABRA2 gene encodes a homeo domain protein required for normal trichome development in *Arabidopsis*. Genes Dev.

[CR65] Rhie A, Walenz BP, Koren S, Phillippy AM (2020). Merqury: reference-free quality, completeness, and phasing assessment for genome assemblies. Genome Biol.

[CR66] Rijpkema AS, Vandenbussche M, Koes R, Heijmans K, Gerats T (2010). Variations on a theme: changes in the floral ABCs in angiosperms. Semin Cell Dev Biol.

[CR67] Roach MJ, Schmidt SA, Borneman AR (2018). Purge Haplotigs: allelic contig reassignment for third-gen diploid genome assemblies. BMC Bioinf.

[CR68] Sader M, Vaio M, Cauz-Santos LA, Dornelas MC, Vieira MLC, Melo N, Pedrosa-Harand A (2021). Large vs small genomes in *Passiflora*: the influence of the mobilome and the satellitome. Planta.

[CR69] Sader MA, Amorim BS, Costa L, Souza G, Pedrosa-Harand A (2019). The role of chromosome changes in the diversification of *Passiflora* L. (Passifloraceae). Syst Biodivers.

[CR70] Sanderson BJ, Wang L, Tiffin P, Wu Z, Olson MS (2019). Sex-biased gene expression in flowers, but not leaves, reveals secondary sexual dimorphism in *Populus balsamifera*. New Phytol.

[CR71] Saul, F., Scharmann, M., Wakatake, T., Rajaraman, S., Marques, A., Freund, M., Bringmann, G., Channon, L., Becker, D., Carroll, E., Low, Y. W., Lindqvist, C., Gilbert, K. J., Renner, T., Masuda, S., Richter, M., Vogg, G., Shirasu, K., Michael, T. P., . . . Fukushima, K. (2023). Subgenome dominance shapes novel gene evolution in the decaploid pitcher plant *Nepenthes gracilis*. Nat Plants. 10.1038/s41477-023-01562-2 37996654

[CR72] Scherzer S, Krol E, Kreuzer I, Kruse J, Karl F, von Ruden M, Escalante-Perez M, Muller T, Rennenberg H, Al-Rasheid KA, Neher E, Hedrich R (2013). The *Dionaea muscipula* ammonium channel *DmAMT1* provides NH_4_^+^ uptake associated with Venus flytrap's prey digestion. Curr Biol.

[CR73] Schiestl FP (2015). Ecology and evolution of floral volatile-mediated information transfer in plants. New Phytol.

[CR74] Scorza LCT, Hernandes-Lopes J, Melo-de-Pinna GFA, Dornelas MC (2017). Expression patterns of *Passiflora edulis APETALA1*/*FRUITFULL* homologues shed light onto tendril and corona identities. Evodevo.

[CR75] Shan H, Cheng J, Zhang R, Yao X, Kong H (2019). Developmental mechanisms involved in the diversification of flowers. Nat Plants.

[CR76] Simao FA, Waterhouse RM, Ioannidis P, Kriventseva EV, Zdobnov EM (2015). BUSCO: assessing genome assembly and annotation completeness with single-copy orthologs. Bioinformatics.

[CR77] Slater GS, Birney E (2005). Automated generation of heuristics for biological sequence comparison. BMC Bioinf.

[CR78] Smith SA, O'Meara BC (2012). treePL: divergence time estimation using penalized likelihood for large phylogenies. Bioinformatics.

[CR79] Soares TL, Jesus ON, Souza EH, Rossi ML, Oliveira EJ (2018). Comparative pollen morphological analysis in the subgenera *Passiflora* and *Decaloba*. An Acad Bras Cienc.

[CR80] Song Y, Wei XQ, Li MY, Duan XW, Sun YM, Yang RL, et al. Nutritional composition and antioxidant properties of the fruits of a Chinese wild *Passiflora foetida*. Molecules. 2018;23(2) 10.3390/molecules23020459 PMC6017921 29463053

[CR81] Souza MM, Palomino G, Pereira TN, Pereira MG, Viana AP (2004). Flow cytometric analysis of genome size variation in some *Passiflora* species. Hereditas.

[CR82] Stamatakis A (2014). RAxML version 8: a tool for phylogenetic analysis and post-analysis of large phylogenies. Bioinformatics.

[CR83] Stanke M, Keller O, Gunduz I, Hayes A, Waack S, Morgenstern B (2006). AUGUSTUS: *ab initio* prediction of alternative transcripts. Nucleic Acids Res.

[CR84] Stritt C, Wyler M, Gimmi EL, Pippel M, Roulin AC (2020). Diversity, dynamics and effects of long terminal repeat retrotransposons in the model grass *Brachypodium distachyon*. New Phytol.

[CR85] Tanaka Y, Sasaki N, Ohmiya A (2008). Biosynthesis of plant pigments: anthocyanins, betalains and carotenoids. Plant J.

[CR86] Tang H, Bowers JE, Wang X, Ming R, Alam M, Paterson AH (2008). Synteny and collinearity in plant genomes. Science.

[CR87] Teo ZWN, Zhou W, Shen L (2019). Dissecting the function of MADS-box transcription factors in orchid reproductive development. Front Plant Sci.

[CR88] Thomson B, Zheng B, Wellmer F (2017). Floral organogenesis: when knowing your ABCs is not enough. Plant Physiol.

[CR89] Wang Y, Tang H, Debarry JD, Tan X, Li J, Wang X, Lee TH, Jin H, Marler B, Guo H, Kissinger JC, Paterson AH (2012). MCScanX: a toolkit for detection and evolutionary analysis of gene synteny and collinearity. Nucleic Acids Res.

[CR90] Wicker T, Sabot F, Hua-Van A, Bennetzen JL, Capy P, Chalhoub B, Flavell A, Leroy P, Morgante M, Panaud O, Paux E, SanMiguel P, Schulman AH (2007). A unified classification system for eukaryotic transposable elements. Nat Rev Genet.

[CR91] Xia Z, Huang D, Zhang S, Wang W, Ma F, Wu B, Xu Y, Xu B, Chen D, Zou M, Xu H, Zhou X, Zhan R, Song S (2021). Chromosome-scale genome assembly provides insights into the evolution and flavor synthesis of passion fruit (*Passiflora edulis* Sims). Hortic Res.

[CR92] Yan S, Bhawal R, Yin Z, Thannhauser TW, Zhang S (2022). Recent advances in proteomics and metabolomics in plants. Mol Hortic.

[CR93] Yotoko KS, Dornelas MC, Togni PD, Fonseca TC, Salzano FM, Bonatto SL, Freitas LB (2011). Does variation in genome sizes reflect adaptive or neutral processes? New clues from *Passiflora*. PLoS One.

[CR94] Zhang RG, Li GY, Wang XL, Dainat J, Wang ZX, Ou S, et al. TEsorter: an accurate and fast method to classify LTR-retrotransposons in plant genomes. Hortic Res. 2022a;9 10.1093/hr/uhac017 PMC9002660 35184178

[CR95] Zhang W, Zhou Q, Lin J, Ma X, Dong F, Yan H, Zhong W, Lu Y, Yao Y, Shen X, Huang L, Zhang W, Ming R (2022). Transcriptome analyses shed light on floral organ morphogenesis and bract color formation in *Bougainvillea*. BMC Plant Biol.

[CR96] Zhang X, Lin S, Peng D, Wu Q, Liao X, Xiang K, Wang Z, Tembrock LR, Bendahmane M, Bao M, Wu Z, Fu X (2022). Integrated multi-omic data and analyses reveal the pathways underlying key ornamental traits in carnation flowers. Plant Biotechnol J.

[CR97] Zhao R, Wang HH, Gao J, Zhang YJ, Li X, Zhou JJ, Liang P, Gao XW, Gu SH (2022). Plant volatile compound methyl benzoate is highly effective against *Spodoptera frugiperda* and safe to non-target organisms as an eco-friendly botanical-insecticide. Ecotoxicol Environ Saf.

