# Supplementary material for: Multi-integrated genomic data for Passiflora foetida provides insights into genome size evolution and floral development in Passiflora
Source: Mol Hortic. 2023 Dec 18;3:27. doi: 10.1186/s43897-023-00076-x (PMC10726625; doi:10.1186/s43897-023-00076-x)
Supplement: Supplementary file 1 — Additional file 1: Fig. S1. Survey of P. foetida genome using kmerfreq and assessment of the final assembly. (a) Density plot shows 17-mer distribution of 25.70 Gb of short reads, indicating a genome size of 456.46 Mb with high repeat content and low heterozygosity. (b) Plots indicates very low contamination using Blobtools. (c) Histogram indicates very few redundant sequences using purge_haplotigs. Fig. S2. Hi-C contact map of the P. foetida genome assembly. Heatmap shows 92.18% of the assembled contigs were anchored into ten pseudochromosomes (LG01 – LG10) with LACHESIS. Fig. S3. GO/KEGG enrichment of expanded and contracted gene families in P. foetida. (a, b) GO enrichment analysis of (a) expanded and (b) contracted gene families in P. foetida. (c, d) KEGG enrichment analysis of (c) expanded and (d) contracted gene families in P. foetida. Fig. S4. One shared recent WGD event between P. foetida and P. edulis. (a) A Ks density plot shows one recent WGD event shared by P. foetida and P. edulis. (b, c) Dot plots show that this recent WGD event was shared by P. foetida and P. edulis. Colors indicate Ks values (sidebar). Fig. S5. TE contents surrounding A-class MADS-box genes in P. foetida and P. edulis. (a) TE contents with ten genes both upstream and downstream of A-class MADS-box genes are shown. (b) A microsyntenic block from LG10.196 to LG10.214 (containing LG10.207/AP1 in P. foetida) was shown. Fig. S6. Samples of P. foetida used for RNA sequencing. (a, b) Photos show a segment of stem of P. foetida in the morning of two successive days. (c) A fully opened flower attracted a honeybee pollinator. (d, e) Photos show (d) dissected parts of floral organs, (e) the stem including a leaf and a tendril (see also panel f). (g-j) Photos show flower buds of different sizes and (j) young fruits, which were all surrounded by persistent glandular bracts. Fig. S7. The numbers of SHEGs in different samples. Heatmap shows the numbers of specifically highly expressed genes (SHEGs) [file 43897_2023_76_MOESM1_ESM.docx]

**Figure S1** Survey of *P. foetida* genome using kmerfreq and assessment of the final assembly.

(a) Density plot shows 17-mer distribution of 25.70 Gb of short reads, indicating a genome size of 456.46 Mb with high repeat content and low heterozygosity. (b) Plots indicates very low contamination using Blobtools. (c) Histogram indicates very few redundant sequences using purge_haplotigs.

**Figure S2** Hi-C contact map of the *P. foetida* genome assembly.

Heatmap shows 92.18% of the assembled contigs were anchored into ten pseudochromosomes (LG01 – LG10) with LACHESIS.

**Figure S3** GO/KEGG enrichment of expanded and contracted gene families in *P. foetida*.

(a, b) GO enrichment analysis of (a) expanded and (b) contracted gene families in *P. foetida*. (c, d) KEGG enrichment analysis of (c) expanded and (d) contracted gene families in *P. foetida*.

**Figure S4** One shared recent WGD event between *P. foetida* and *P. edulis*.

(a) A *Ks* density plot shows one recent WGD event shared by *P. foetida* and *P. edulis*. (b, c) Dot plots show that this recent WGD event was shared by *P. foetida* and *P. edulis*. Colors indicate *Ks* values (sidebar).

**Figure S5** TE contents surrounding A-class MADS-box genes in *P. foetida* and *P. edulis*.

(a) TE contents with ten genes both upstream and downstream of A-class MADS-box genes are shown. (b) A microsyntenic block from LG10.196 to LG10.214 (containing LG10.207/AP1 in *P. foetida*) was shown.

**Figure S6** Samples of *P. foetida* used for RNA sequencing.

(a, b) Photos show a segment of stem of *P. foetida* in the morning of two successive days. (c) A fully opened flower attracted a honeybee pollinator. (d, e) Photos show (d) dissected parts of floral organs, (e) the stem including a leaf and a tendril (see also panel f). (g-j) Photos show flower buds of different sizes and (j) young fruits, which were all surrounded by persistent glandular bracts.

**Figure S7** The numbers of SHEGs in different samples.

Heatmap shows the numbers of specifically highly expressed genes (SHEGs) for each sample group. Values are the numbers of upregulated DEGs of one sample (vertical) versus another (horizontal). Abbreviations: Br, bracts; Se, sepals; Pe, petals; Ri, radii; Pa, pali; Ag, androgynophore; St, stamens; and Pi, pistils.

**Figure S8** GO enrichment of SHEGs in different samples.

Abbreviations of GO function classifications: BP, biological process; CC: cellular component; MF, molecular function. The x-axis indicates gene ratio.

**Figure S9** KEGG enrichment of SHEGs in different samples.

The x-axis in each subfigure indicates the number of enriched DEGs.

**Figure S10** GO/KEGG enrichment of upregulated DEGs in Pi and St.

(a) GO and (b) KEGG enrichment analysis of pistils (Pi). (c) GO and (d) KEGG enrichment analysis of stamens (St). The x-axes in panels b and d indicate the number of enriched DEGs.

**~~Figure S11~~** ~~Volatile compounds in~~ *~~P. foetida~~* ~~flowers.~~

~~(a-b) GC peaks for three replicates of (a) bracts and (b) the remaining parts of~~ *~~P. foetida~~* ~~flowers. Volatile compounds were identified by GC-MS. The peaks were compared against NIST library using Xcalibur software. Names of compounds (black integer labels in the graphs) were shown on the right.~~

**Figure S11** The expression levels of biosynthetic genes of major floral volatiles in *P. foetida*.

Heatmaps show the expression levels of methyl benzoate biosynthetic genes in different floral whorls of *P. foetida*. The genes were identified by BLAST searches. Abbreviations in a: PAL, phenylalanine ammonia-lyase; PXA1, peroxisomal ATP-binding cassette transporter 1; CNL, cinnamoyl-CoA ligase; CHD, cinnamoyl-CoA hydratase/dehydrogenase; KAT, 3-ketoacyl CoA thiolase; TE, thioesterase; BSMT, benzoic acid/salicylic acid carboxyl methyltransferases; and BALD, benzaldehyde dehydrogenase.
